# Chromatin accessibility and transcription dynamics during in vitro astrocyte differentiation of Huntington’s Disease Monkey pluripotent stem cells

**DOI:** 10.1186/s13072-019-0313-6

**Published:** 2019-11-13

**Authors:** Alexandra V. Goodnight, Isaac Kremsky, Sujittra Khampang, Yoon Hee Jung, James M. Billingsley, Steven E. Bosinger, Victor G. Corces, Anthony W. S. Chan

**Affiliations:** 1 Division of Neuropharmacology and Neurologic Diseases, Yerkes National Primate Research Center, Atlanta, GA 30322 USA; 2 Department of Human Genetics, Emory University, Atlanta, GA 30322 USA; 3 Genetics and Molecular Biology Program, Graduate Division of Biological and Biomedical Sciences, 1462 Clifton Rd, Atlanta, GA 30322 USA; 4 Embryonic Stem Cell Research Center, School of Biotechnology, Suranaree University of Technology, Nakhon Ratchasima, Thailand; 5 Division of Microbiology and Immunology, Yerkes National Primate Research Center, Emory University, Atlanta, GA USA; 6 Department of Pathology and Laboratory Medicine, Emory University, Atlanta, GA USA

**Keywords:** Neurodegeneration, Neural progenitor cells, Glia, Brain, ATAC-seq

## Abstract

**Background:**

Huntington’s Disease (HD) is a fatal neurodegenerative disorder caused by a CAG repeat expansion, resulting in a mutant huntingtin protein. While it is now clear that astrocytes are affected by HD and significantly contribute to neuronal dysfunction and pathogenesis, the alterations in the transcriptional and epigenetic profiles in HD astrocytes have yet to be characterized. Here, we examine global transcription and chromatin accessibility dynamics during in vitro astrocyte differentiation in a transgenic non-human primate model of HD.

**Results:**

We found global changes in accessibility and transcription across different stages of HD pluripotent stem cell differentiation, with distinct trends first observed in neural progenitor cells (NPCs), once cells have committed to a neural lineage. Transcription of p53 signaling and cell cycle pathway genes was highly impacted during differentiation, with depletion in HD NPCs and upregulation in HD astrocytes. E2F target genes also displayed this inverse expression pattern, and strong associations between E2F target gene expression and accessibility at nearby putative enhancers were observed.

**Conclusions:**

The results suggest that chromatin accessibility and transcription are altered throughout in vitro HD astrocyte differentiation and provide evidence that E2F dysregulation contributes to aberrant cell-cycle re-entry and apoptosis throughout the progression from NPCs to astrocytes.

## Background

Huntington’s disease (HD) is an autosomal dominant, neurodegenerative disease characterized by progressive brain atrophy, along with cognitive and motor dysfunction, and affecting adults between 35 and 55 years of age [1]. Although several potential therapeutic strategies have been identified, no cure or therapies exist to prevent or reverse disease progression [2–7]. HD results from an expansion of more than 40 CAG repeats in exon 1 of the huntingtin gene, *HTT*, which gives rise to a mutant huntingtin protein (mHTT) with an extended polyglutamine (polyQ) tract at the N-terminus [8–13]. mHTT can form aggregates and nuclear inclusions, a signature characteristic of HD [9, 12, 14], and can be cleaved to create polyQ-containing protein fragments [15].

The disruption of many cellular processes, including neurodevelopment, cell cycle, apoptosis, mitochondrial function, inflammation, and synapse formation and activity by mHTT in neurons is well documented, but incompletely understood [16–21]. However, transcriptional dysregulation is thought to play an important role in driving these pathogenic mechanisms [16, 18–21]. Altered transcriptional profiles are evident very early in HD and impact neurogenesis, setting the stage for dysfunctional homeostasis and degeneration in the adult brain [16, 18–20, 22, 23]. Furthermore, the impact of mHTT on transcription varies between neural populations in the central nervous system (CNS), as well as across development [17, 21, 24–28]. It has been demonstrated that mHTT aberrantly interacts with and sequesters critical proteins, such as transcription factors (TFs) and enzymes involved in epigenetic processes, causing widespread changes in transcription and disruption of important cellular processes in the brain [1, 8, 29–37]. For example, mHTT binds to p53, altering transcriptional activity of select p53-target genes [38, 39]. p53 regulates a diverse range of specific cellular processes, including cell cycle, apoptosis, DNA repair, metabolism, and mitochondrial function [40, 41]. However, the downstream transcriptome consequences of the mHTT interactions with TFs, such as p53, are not well understood.

The transcriptome is largely regulated by epigenetic mechanisms, such as binding of TFs and architectural proteins, histone modifications, and DNA methylation. The accessibility (relatively high DNAse-seq or ATAC-seq signal) of genomic regulatory elements, such as promoters and enhancers, reflects the binding of TFs and architectural proteins, and thus indicates regions regulated by the activation and repression of transcription [42]. Accessibility is highly specific between cell types, developmental timepoints, and pathological conditions [43], and although there have been no investigations of chromatin accessibility dynamics in HD, there is growing support that epigenetic mechanisms contribute to HD transcriptional dysregulation and pathology [30, 39, 44–54]. Thus, studies exploring accessibility at specific developmental stages and in specific cell types, and its relationship to transcriptional changes, have the potential to contribute greatly to our understanding of HD, both in terms of the mechanisms involved as well as in the discovery of HD therapies.

Most studies of transcriptional and epigenetic dysregulation in HD pathogenesis have focused on neuronal models and heterogenous brain tissues. However, neurons require glia cells, which comprise 90% of the human CNS cell population, for normal function, homeostasis, and the establishment and healthy activity of neural circuits [55–58]. Given its ubiquitous expression and vast impact on cellular processes, it is undeniable that mHTT affects all neural cell types, not just certain neuron populations, yet little is known about its impact on glial development and function in HD.

Astrocytes are the most abundant type of glial cell and are critical CNS regulators, with roles in neurodevelopment, neuronal metabolic support, synaptic formation and function, tissue repair, neuroprotection, ion signaling homeostasis, and complex brain functions such as sleep, memory, and breathing [56, 57, 59–63]. Given this, it is not surprising that a growing body of evidence suggests a role for astrocytes in HD pathology [64–74]. Both mHTT expression and aggregate formation have been reported in HD astrocytes [70, 72, 75, 76]. Increased expression of glial fibrillary acidic protein (*GFAP*), a marker for astrogliosis, is a well-documented indicator of early neural damage in HD [17, 67, 70, 77–80]. Interestingly, HD neurons had improved molecular phenotypes when co-cultured with WT astrocytes, demonstrating that functional astrocytes can protect against HD-mediated neurotoxicity [70]. In contrast, WT neurons co-cultured with HD astrocytes showed markers of early neurodegeneration, which could be reversed through chemical inhibition of astrocytic glutamate receptors, suggesting that HD astrocytes cause intercellular dysregulation that may contribute to HD pathogenesis and serve as a potential therapeutic target for HD treatment [64, 70].

While astrocyte dysfunction is gaining growing recognition for its role in HD pathogenesis [65, 70], most studies examining HD astrocytes have used rodent models. However, it has been established that primate and rodent astrocytes have morphological and functional differences [81–83]. Here, we examined the impact of mHTT on chromatin accessibility and transcription during in vitro astrocyte differentiation using a transgenic non-human primate Rhesus macaque (*Macaca mulatta*) model of HD [78, 84–88]. Given similarities in CNS anatomy, neurodevelopment, and behavior, Rhesus may offer a more translatable model for investigating neurological disease pathology and identifying efficient therapies.

We used induced pluripotent stem cells (iPSCs) derived from transgenic HD Rhesus monkeys to establish stable NPC lines, which are able to differentiate into functional neurons and astrocytes that recapitulate HD phenotypes [2, 89–91]. RNA sequencing (RNA-seq) and Assay for Transposase-Accessible Chromatin using sequencing (ATAC-seq) experiments were performed in parallel, in HD and WT Rhesus macaque cell lines, to characterize chromatin accessibility dynamics and transcriptome profiles during HD astrocyte differentiation. This discovery-based approach revealed genome-wide alterations in chromatin accessibility and transcription in HD cells relative to WT across differentiation, with observable trends occurring once cells have committed to a neural lineage (NPCs through astrocytes). Although alterations in accessibility at promoters were evident in HD cells across differentiation, most differences in accessibility occurred distal to promoters. At a subset of putatively active Rhesus macaque brain enhancers, the altered chromatin accessibility observed in HD astrocytes appeared to be established in the NPC stage. In addition, an integrated analysis of RNA-seq and ATAC-seq results revealed consistent mHTT-induced deficits in various cell-cycle-related pathways, such as p53 signaling and E2F target genes, as well as a striking correlation between changes in gene expression and TF occupancy at putative enhancers. Taken together, these results suggest a pathogenic mechanism in HD astrogenesis: dysregulation of several interacting pathways causes expression signatures indicating delayed cell-cycle progression in HD NPCs and aberrant cell-cycle re-entry and apoptosis, possibly through an E2F1-p53-dependent mechanism in astrocytes.

## Results

### In vitro astrocyte differentiation of HD and WT Rhesus macaque cells

Stable NPC lines were previously established from HD and WT Rhesus macaque pluripotent stem cells (PSCs) [89]. HD cell lines carried a construct for exon 1 of the human *HTT* gene with 65 CAG repeats, along with an additional GFP vector, both under the regulation of the human polyubiquitin-C (*UBC*) promoter [88]. NPC cell cultures were induced to differentiate into astrocytes using a previously established 30-day protocol [92] (Additional file 1: Figure S1a). Quantitative reverse transcription PCR (RT-qPCR) analysis of both cell lines across astrocyte differentiation showed that endogenous *HTT* is expressed at each stage (Additional file 1: Figure S1b). HD cells showed increased expression of exon 1 of the *HTT* transcript relative to exon 26 compared to WT cells, demonstrating expression of the *mHTT* transgene in HD cells (Additional file 1: Figure S1c). In addition, both HD and WT cell lines exhibit appropriate, stage-specific expression of canonical markers over the course of differentiation, such as *OCT4* and *SOX2* in PSCs (Additional file 1: Figure S1d, e); *SOX2*, *MSI1*, and *NES* in NPCs (Additional file 1: Figure S1d, e); and *GFAP*, *APOE*, and *LCN2* in astrocytes (Additional file 1: Figure S1f, g). Neuronal (*MAP2, TH, GAD*), microglia (*CX3CR1*), and oligodendrocyte (FOXO4) markers were largely downregulated following astrocyte differentiation (Additional file 1: Figure S1 h–j).

### Altered transcription profiles are observed during in vitro astrocyte differentiation in cells expressing mHTT

We performed RNA-seq in HD and WT Rhesus macaque cells at four timepoints across astrocyte differentiation: PSCs, NPCs, and 3 days after NPCs were induced into astrocyte differentiation (day 3) and astrocytes (day 30) (Additional file 1: Figure S1a). RNA-seq data for markers of PSCs and NPCs (Additional file 1: Figure S2a, b), astrocytes (Additional file 1: Figure S2c, d), and other neural cell types (Additional file 1: Figure S2e, f) generally reflect the trends observed by RT-qPCR (Additional file 1: Figure S1d, i). Some minor inconsistencies are observed, but this is to be expected due to the high degree of noise inherent in RNA-seq data. Interestingly, the inclusion of day 3 expression data in RNA-seq experiments revealed that HD cells show early expression of astrocyte markers (*GFAP, APOE, LCN2*) compared to WT cells (Additional file 1: Figure S2c, d). These findings are in agreement with results from a recent study, showing that heterogeneous neural cultures (NPCs, astrocytes, and neurons) derived from human HD iPSCs exhibit altered expression of neurodevelopmental pathway genes compared to WT cells [25].

**Fig. 1:**
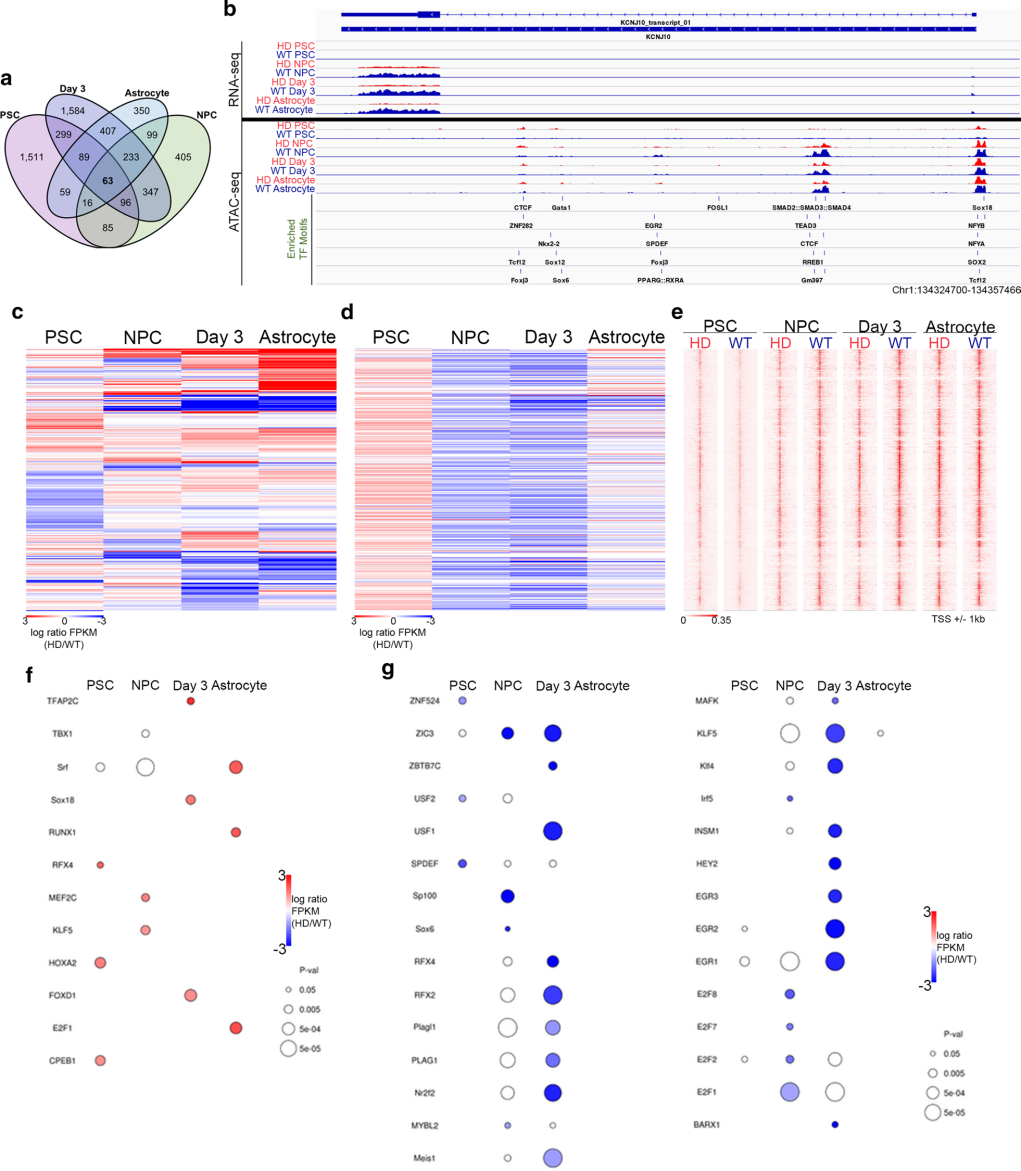
Characterization of promoter-proximal THSSs at differentially expressed genes during HD astrocyte differentiation. **a** Venn diagram showing overlap of genes differentially expressed between HD and WT cells at each stage of astrocyte differentiation. **b** Track view of RNA-seq and THSSs, as well as identified motifs within ATAC-seq peaks across the *KCNJ10* gene. HD samples are shown in red and WT samples are shown in blue. **c** Heatmap depicting 5643 genes found DE at any stage of differentiation. Red indicates increased expression in HD cells and blue indicates reduced expression in HD cells. Each row corresponds to the same gene. **d** Heatmap depicting differential THSS enrichment at DE gene promoters. The red color represents HD enrichment and the blue color indicates HD depletion. Genes arranged according to gene order in **c**. **e** Distributions of ATAC-seq peaks around the promoter (± 2 kb TSS) in HD and WT cells at each timepoint across differentiation. **f**, **g** TF motifs identified at differential promoter-proximal ATAC-seq peaks in HD (**f**) and WT (**g**) cells. Only TF motifs with significant enrichment at least at one stage were included (*p* < 0.05)

Principal component analysis (PCA) of log2-transformed normalized RNA-seq data demonstrates that the least variability between HD and WT cells occurs at the PSC stage, with progressive divergence at each stage of differentiation and the greatest variability takes place at the astrocyte stage (Additional file 1: Figure S3a). In addition, global log2-transformed normalized read counts illustrate the distinct expression patterns of HD and WT cell lines that become progressively dissimilar as cells progress towards the astrocyte stage (Additional file 1: Figure S3b). Overall, RNA-seq data segregated by cell type (HD vs. WT) and stage (PSCs, NPCs, day 3, astrocytes), with low variability between replicates. This demonstrates that the observed variance is a result of mHTT and stage of astrocyte differentiation. Cuffdiff [93] analysis of the RNA-seq data uncovered 5643 differentially expressed (DE) genes between HD and WT cells in at least one timepoint (*q* < 0.05), with 2218 DE genes in PSCs, 1344 in NPCs, 3118 at day 3, and 1316 in astrocytes (Fig. 1a; Table 1; Additional file 2: Table S2). Of these, 63 genes are differentially expressed across all 4 stages (Fig. 1a; Additional file 3: Table S3). Figure 1b shows an example DE gene, *KCNJ10* that has decreased expression in HD astrocytes compared to WT astrocytes, with differences observed as early as the NPC stage. *KCNJ10*, which encodes the Kir4.1 potassium ion channel, has been previously demonstrated to be downregulated in HD mouse astrocytes [72]. Thus, these data provide further support for this finding, as well as for the ability of Rhesus macaque astrocytes to recapitulate reported HD phenotypes.

**Table 1:** Number of DE genes at each stage of astrocyte differentiation

	Total DE	Up in HD	Down in HD
PSC	2218	881 (39.7%)	1337 (60.3%)
NPC	1344	618 (46%)	726 (54%)
Day 3	3118	1405 (45.1%)	1713 (54.9%)
Astrocyte	1316	729 (55.4%)	587 (44.6%)

We next compared the DE genes identified in the RNA-seq analysis to those differentially expressed in heterogeneous neural cultures derived from human HD iPSCs [25]. Only DE genes annotated in the genomes of both species were counted. This comparison yielded 824 overlapping genes (*p* = 3.14e−57), which is more than the reported overlap with a mouse data set [25] (Additional file 1: Figure S3c). It is not surprising that 944 DE genes found by the HD iPSC Consortium were not found to be differentially expressed here. Differences in cell types cultured between their experiments and ours (a high proportion of neurons vs. highly homogenous astrocytes and their precursors) will a priori preclude any neuron-specific DE genes from our study that are in theirs; however, critical differences between the HD iPSC consortium’s experimental protocol and ours (physiological expression of the full mHTT transcript vs. overexpression of exon 1 of mHTT) could contribute to any discrepancies that occur in astrocytes. Regardless, the sizeable, statistically significant, overlap in DE genes between the HD iPSC Consortium’s study and ours suggests that our system is capable of recapitulating many of the changes in molecular phenotype that occur due to mHTT expression in a physiological context. On the other hand, we identified 4534 DE genes that were not identified by the HD iPSC Consortium [25]. Moreover, a similar comparison of our DE gene list to a list of 3853 DE genes in HD human brain tissues demonstrated 1323 genes shared between the two data sets (*p* = 3.13e−12; Additional file 1: Figure S3d) [17]. As with the comparison to the in vitro DE gene list, we identified 4035 DE genes not found by Labadorf et al. [17], further supporting the hypothesis that we are able to identify more astrocyte-specific DE genes using homogenous cell populations. In all, we found 2364 DE genes in the NPC through astrocyte stages that were not found before, suggesting that we have identified many astrocyte-specific genes that are altered due to mHTT and that have not been previously identified in genome-wide primate studies of HD.

Hierarchical clustering of all DE genes shows associations between NPC, day 3 and astrocyte samples, whereas PSCs show a unique DE profile (Fig. 1c). Taken together, these findings provide evidence that the Rhesus macaque HD model recapitulates transcriptome phenotypes reported from in vivo and in vitro human HD models, provides greater resolution and statistical power for high-throughput studies of NPCs and astrocytes, and identifies a set of DE genes potentially involved in either HD progression or attenuation that were not identified in previous high-throughput studies of mixed cell populations.

### mHTT expression induces dynamic alterations in promoter-proximal chromatin accessibility during astrocyte differentiation

To characterize genome-wide changes in chromatin accessibility and assess their association with the altered transcriptome in HD, we performed ATAC-seq [94] on HD and WT cells in parallel with RNA-seq experiments. ATAC-seq was performed in 2 replicates, displaying high correlation in all samples, and thus, replicates were combined and various quality control steps were applied (Additional file 1: Figure S3d, e; Additional file 4: Table S4).

We isolated sub-nucleosomal fragments (< 125 bp), which correspond to genomic regions protected from the Tn5 transposase by proteins bound to the DNA (Tn5 hypersensitive sites [THSSs]). Overall, there were 2467 differential promoter-proximal THSSs (315 HD enriched, and 2152 HD depleted; Table 2), and 44,649 differential distal THSSs (30,588 HD enriched, and 14,061 HD depleted; Table 2), suggesting that although genomic changes in chromatin accessibility occur, HD cells demonstrate an overall depletion of promoter accessibility during differentiation of PSCs into astrocytes. Of note, no differential promoter-proximal THSS enrichment occurs at the *KCNJ10* locus, but differences in intragenic distal THSSs are evident between HD and WT cells during differentiation and coincide with gene expression changes at each stage (Fig. 1b).

**Table 2:** Number of differential ATAC-seq peaks at each stage of differentiation

	HD THSSs (proximal)	HD THSSs (distal)	Total differential THSSs	HD-enriched differential THSSs	HD-depleted differential THSSs
PSC	13,127	75,937	2676	1923 (71.9%)	753 (28.1%)
NPC	13,345	128,751	16,497	9437 (57.2%)	7060 (42.8%)
Day 3	13,456	150,929	20,883	13,201 (63.2%)	7682 (36.8%)
Astrocyte	13,677	152,396	7060	6342 (89.8%)	718 (10.2%)

To examine the genome-wide relationship between gene expression and promoter accessibility during astrocyte differentiation in the presence of mHTT expression, we first arranged differential promoter-proximal THSS enrichment at each stage according to the order of DE genes, as shown in Fig. 1c, d. Heatmaps of the distribution of THSS enrichment around the TSS shows that differential peaks are enriched in signal in the appropriate condition (HD or WT) in each stage (Fig. 1e). Overall, changes in proximal THSSs only correlate weakly with differential expression (Additional file 1: Figure S3f, g).

Next, a motif analysis of differential proximal THSSs was performed. More motifs were identified for HD depleted than HD-enriched proximal THSSs (Fig. 1f, g; Additional file 5: Table S5), consistent with the ratio of HD-up/HD-down differential THSSs observed. Accessibility changes at enriched motifs occur in the same direction as changes in corresponding TF expression at many proximal THSSs across differentiation, and in most cases, the accessibility changes preceded changes in gene expression (Fig. 1f, g). This suggests that, broadly speaking, alterations in TF binding across HD astrocyte differentiation may be responsible for changes in gene expression, though further studies are needed to confirm thishypothesis and to demonstrate a causal link.

Interestingly, two TF motifs were found to be differentially enriched between HD and WT cells in opposite directions at different stages: RFX4 and E2F1 (Fig. 1f, g). In PSCs, RFX4 is HD-enriched; however, NPC and day 3 samples show WT enrichment of this motif. The RFX2 motif shows similar patterns of depletion in NPCs and at day 3 (Fig. 1g). In addition, motif accessibility and expression of E2F1 are WT-enriched in NPCs and HD-enriched in astrocytes. It is notable that E2F2, E2F7, and E2F8 are also WT-enriched in NPCs (Fig. 1g).

Taken together, the analysis of promoter-proximal THSSs showed stage-specific motif enrichment and gene expression patterns, suggesting that mHTT expression could result in highly dynamic alterations during astrocyte differentiation. Although the majority of gene expression changes cannot simply be explained by corresponding changes in accessibility at promoters at the developmental stage in which gene expression changes occur, clear alterations of promoter accessibility were observed that coincide with mHTT-induced gene expression changes.

### Extensive alterations in THSS accessibility at distal loci genome-wide occur during astrocyte differentiation in the presence of mHTT

**Fig. 2:**
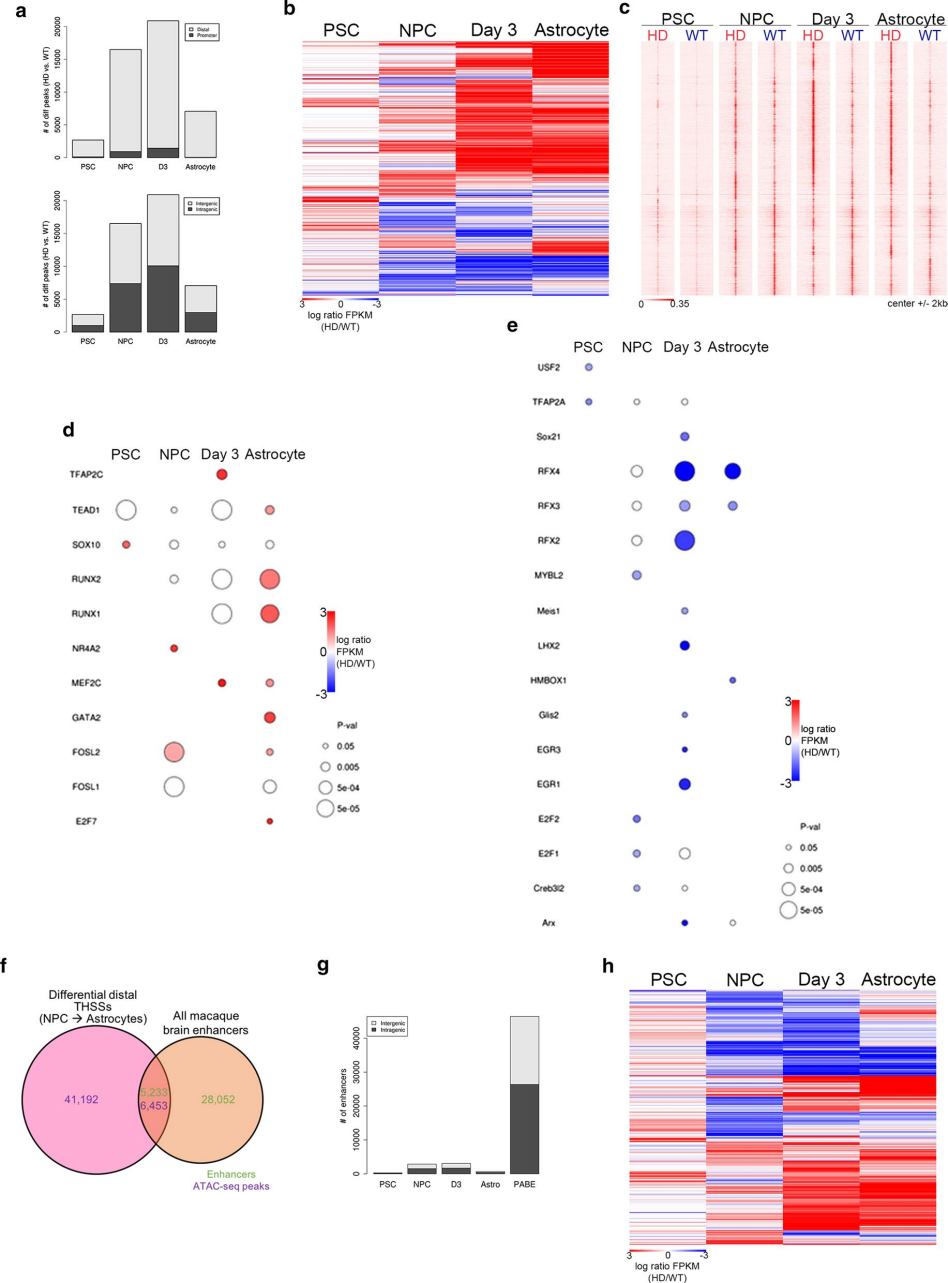
Characterization of differential distal THSSs during HD astrocyte differentiation. **a** Genome-wide distribution of differential THSSs between HD and WT cells across differentiation identified from ATAC-seq non-nucleosomal fragments. Both distal vs. proximal (top) and intergenic vs. intragenic distributions (bottom) are reported. THSSs located ± 500 nt from a TSS are considered proximal. **b** Heatmap depicting differential enrichment of distal ATAC-seq THSSs at each stage of differentiation. The red color represents HD enrichment and the blue color indicates HD depletion. **c** Genome-wide distributions of distal ATAC-seq THSSs from HD and WT cells at each timepoint. **d**, **e** TF motifs identified from distal ATAC-seq peaks that were enriched in HD (D) and WT (E) cells. Only TF motifs with significant enrichment at least at one stage were included (*p* < 0.05). **f** Venn diagram showing overlap of differential distal ATAC-seq THSSs and Putative Active Brain Enhancers (PABEs) previously published [43]. Purple denotes ATAC-seq peaks and green indicates macaque brain enhancers. **g** Genome-wide distribution of differential enhancer THSSs across HD astrocyte differentiation. **h** Heatmap depicting differential enrichment of distal ATAC-seq THSSs at PABEs during differentiation. The red color represents HD enrichment and the blue color indicates HD depletion

The genome-wide distribution of differential THSSs at each stage showed that most differences occurred distal to promoters (> 500 bp from any TSS): 96% in PSCs, 95% in NPCs, 93% in day 3, and 99% in astrocytes (Fig. 2a). However, differential THSSs were more evenly distributed between intergenic and intragenic regions; 37% in PSCs, 45% in NPCs, 48% in day 3, and 42% in astrocytes were intragenic (Fig. 2a). To gain insights into alterations in distal chromatin accessibility across HD astrocyte differentiation, hierarchical clustering was performed on distal THSSs that showed differential enrichment in at least one stage (Fig. 2b). Scaled distal THSS reads for each sample are shown and demonstrate that the differential regions are indeed enriched for the appropriate signal (Fig. 2c). Distinct clusters of distal THSSs occur between HD and WT, especially in NPCs, day 3, and astrocytes (Fig. 2b, c).

Distal THSSs were strikingly similar between HD and WT in PSCs (Fig. 2b, c); alterations in distal accessibility first appear in NPCs, when cells are still multipotent but have committed to a neural lineage. Given the body of research providing evidence for the early dysregulation of a myriad of neurodevelopmental pathways in HD [16, 25, 44], this finding suggests that the neurodevelopmental dysregulation in HD may be established, at least in part, by alterations in distal TF binding as early as the NPC stage. Future research in which TF binding is modulated prior to differentiation in HD NPCs is necessary to confirm this hypothesis.

Next, we performed a motif enrichment analysis of differential distal THSSs (Fig. 2d, e). Several distal motifs show similar enrichment patterns to proximal THSSs (Fig. 1f, g; Additional file 5: Table S5). For example, distal WT-enrichment of RFX2, RFX3, and RFX4 motifs occurs in NPCs and astrocytes and corresponded with DE of these TFs (Figs. 1f, 2e). E2F1 and E2F2 motifs also show moderate distal WT-enrichment in NPCs, while the E2F7 motif is enriched in HD astrocytes (Fig. 2d). In addition, of note is the enrichment of the FOSL2 motif at distal THSSs in HD NPCs and astrocytes, with corresponding expression changes (Fig. 2d). Our results suggest that changes in distal TF occupancy are widespread and involve a wide range of TFs.

### Enhancer accessibility is altered due to mHTT expression during in vitro astrocyte differentiation

The finding that differential distal THSS enrichment during HD differentiation is established in the NPC stage (Fig. 2b, c), after committing to a neural lineage, raises the question of whether these differential distal THSSs are occurring at neurodevelopmental and astrocyte-specific enhancers. To investigate this, we utilized published H3K27ac chromatin immunoprecipitation with sequencing (ChIP-seq) data from a study that identified putative active brain enhancers (PABEs) in macaque tissues [43]. Since we used the MacaM reference genome for all our analyses, we realigned the raw ChIP-seq data to the MacaM reference genome and followed the protocol of Vermut et al. to call macaque enhancers [43, 95].

We identified 33,285 PABEs in MacaM; of those 5233 overlapped with 6453 of our differential distal THSSs (Fig. 2f). All stages show similar ratios of differential intragenic/(intragenic + intergenic) enhancers to each other and to total PABEs: 53% in PSCs, 53% in NPCs, 55% in day 3, 62% in astrocytes; and 56% for all PABEs) (Fig. 2g). Hierarchical clustering of differential THSS enrichment at PABEs shows distinct clusters of altered enhancer accessibility, beginning in NPCs and persisting through day 3 and astrocyte samples (Fig. 2h), similar to what was observed for all differential distal THSSs (Fig. 2b). Again, PSCs show the least differences between HD and WT accessible PABEs (Fig. 2h), further supporting the idea that the mHTT-induced alterations in TF binding in mature HD cells are established in the NPC stage.

**Fig. 3:**
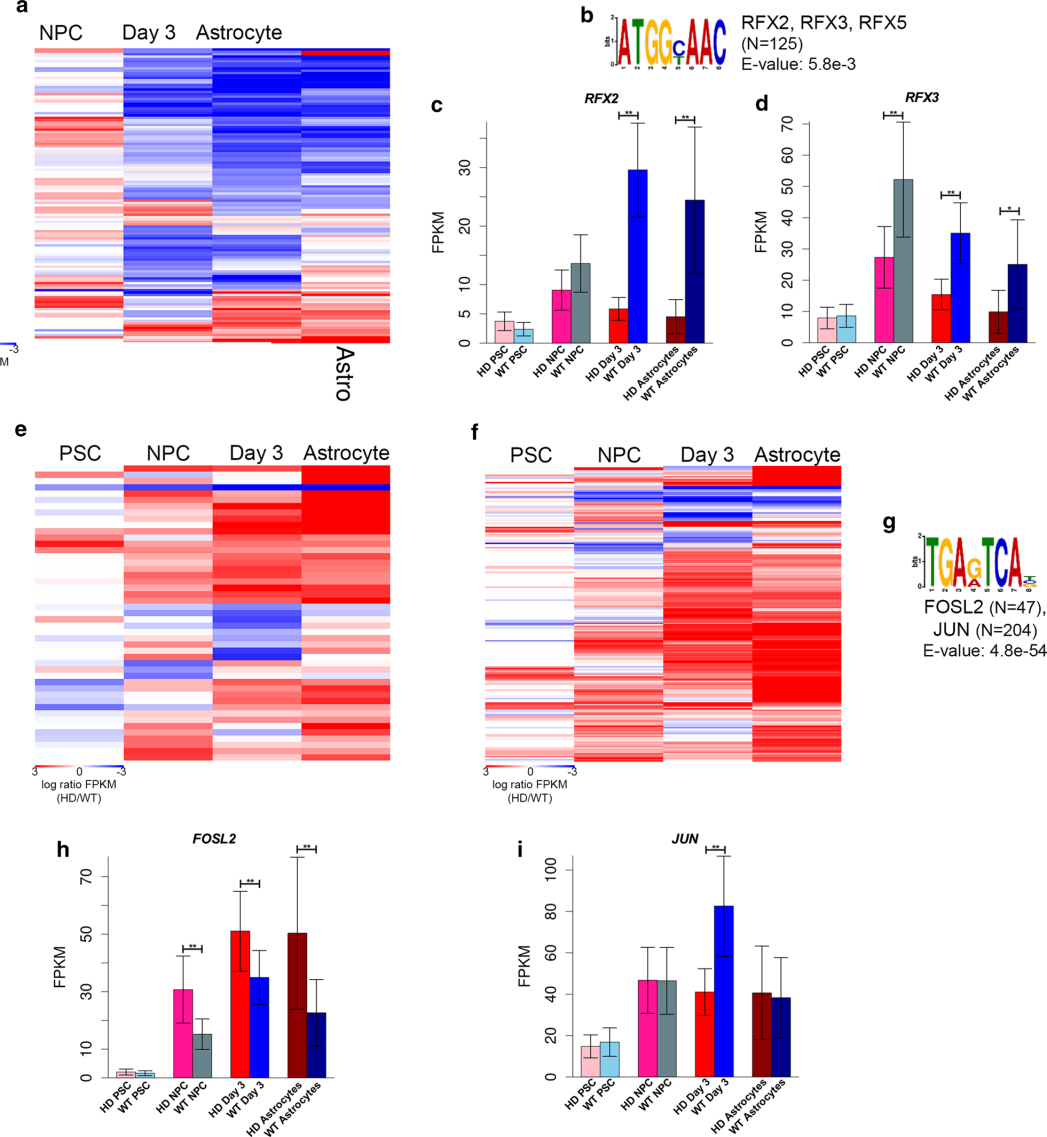
Differences in TF-binding site accessibility occur in putative enhancers at every stage of differentiation in HD cells. **a** Heatmap depicting differential ATAC-seq signal in HD and WT cells at macaque brain enhancers containing the RFX2, RFX3, and RFX5 binding sequences (*N* = 125). The red color represents enrichment and the blue color indicates depletion of THSSs containing this RFX motif in HD cells. **b** RFX2, RFX3, and RFX5 motif found by de novo motif analysis at enhancers showing differential ATAC-seq. **c**, **d** Bar graphs generated from RNA-seq data depicting differential expression of *RFX2* (**c**) and *RFX3* (**d**) showing differential expression corresponding with motif accessibility. Average FPKM for each sample was plotted. Error bars show 95% confidence intervals (**p < 0.001 and *p < 0.01, differential expression analysis). **e**, **f** Heatmap depicting differential THSSs at macaque brain enhancers containing FOSL2 (*N* = 47; **e**) and JUN (*N* = 204; **f**) binding sequences. Red represents HD enrichment and blue represents HD depletion. **g** The motif for FOSL2 and JUN found at enhancers showing differential ATAC-seq enrichment. **h, i** Bar graphs generated from RNA-seq for *FOSL2* (**h**) and *JUN* (**i**) expression across differentiation. Error bars show 95% confidence intervals (**p < 0.001 and *p < 0.01, differential expression analysis)

Next, MEME-ChIP [96] was used to perform a de novo motif discovery analysis at PABEs that overlap differential THSSs at any stage, revealing significant enrichment of several TF motifs (Fig. 3; Additional file 1: Figure S4). RFX2, RFX3, and RFX4 motifs are WT-enriched (Fig. 2e). However, in astrocytes, this motif was observed to be HD-enriched at some PABEs and WT-enriched at others (Fig. 3a, b). Previous research has described tissue-specific expression profiles for the RFX TFs [97]. RFX2 is best known for its role in regulating spermiogenesis, where reduced expression induced apoptosis; it is also highly expressed in the brain [97, 98], while RFX3 is highly expressed in fetal tissues and the brain [97]. Consistent with these findings, we observed DE of RFX3 (Fig. 3d) in NPCs and at day 3, at earlier stages of differentiation, and DE of RFX2 (Fig. 3c) in day 3 and astrocyte samples. RFX4 expression followed similar trends to that of RFX2, with reduced expression at day 3 and in HD astrocytes (Additional file 1: Figure S4a). RFX5, which is reported to have broad expression profiles across a variety of tissues [97], was only DE in PSCs (Additional file 1: Figure S4b). Overall, RFX motif accessibility is altered across HD astrocyte differentiation.

In addition, the FOSL2 motif shows increasing enrichment at HD-accessible PABEs (*N* = 47; *E* value = 4.8e−54) from the NPC to astrocyte stages (Fig. 3e, g), which is consistent with the observed distal enrichment of the FOSL2 motif in HD NPCs and astrocytes (Fig. 2d). DE of FOSL2 corresponded with increased motif enrichment at HD-accessible enhancers (Fig. 3h). FOSL2 is a member of the AP-1 TF complex, along with JUN, which is also enriched in HD-accessible PABEs across differentiation (*N* = 204; *E* value = 4.8e−54) (Fig. 3f, g). However, DE of JUN is seen at day 3 of astrocyte differentiation and does not correspond with differential motif accessibility at PABEs (Fig. 3f, i). Our results identify several protein families, including RFX and FOS/JUN, that are altered in our HD model and may play a role in astrocyte HD pathology via altered binding to enhancers. While ChIP-seq and similar studies are needed to verify exactly which proteins are involved, our study provides a list of likely candidates and suggests that mHTT expression induces alterations in TF binding at enhancers containing these motifs in astrocytes and their precursor cells in vitro.

### Gene Ontology (GO) analysis of DE genes across astrocyte differentiation in the presence of mHTT

**Fig. 4:**
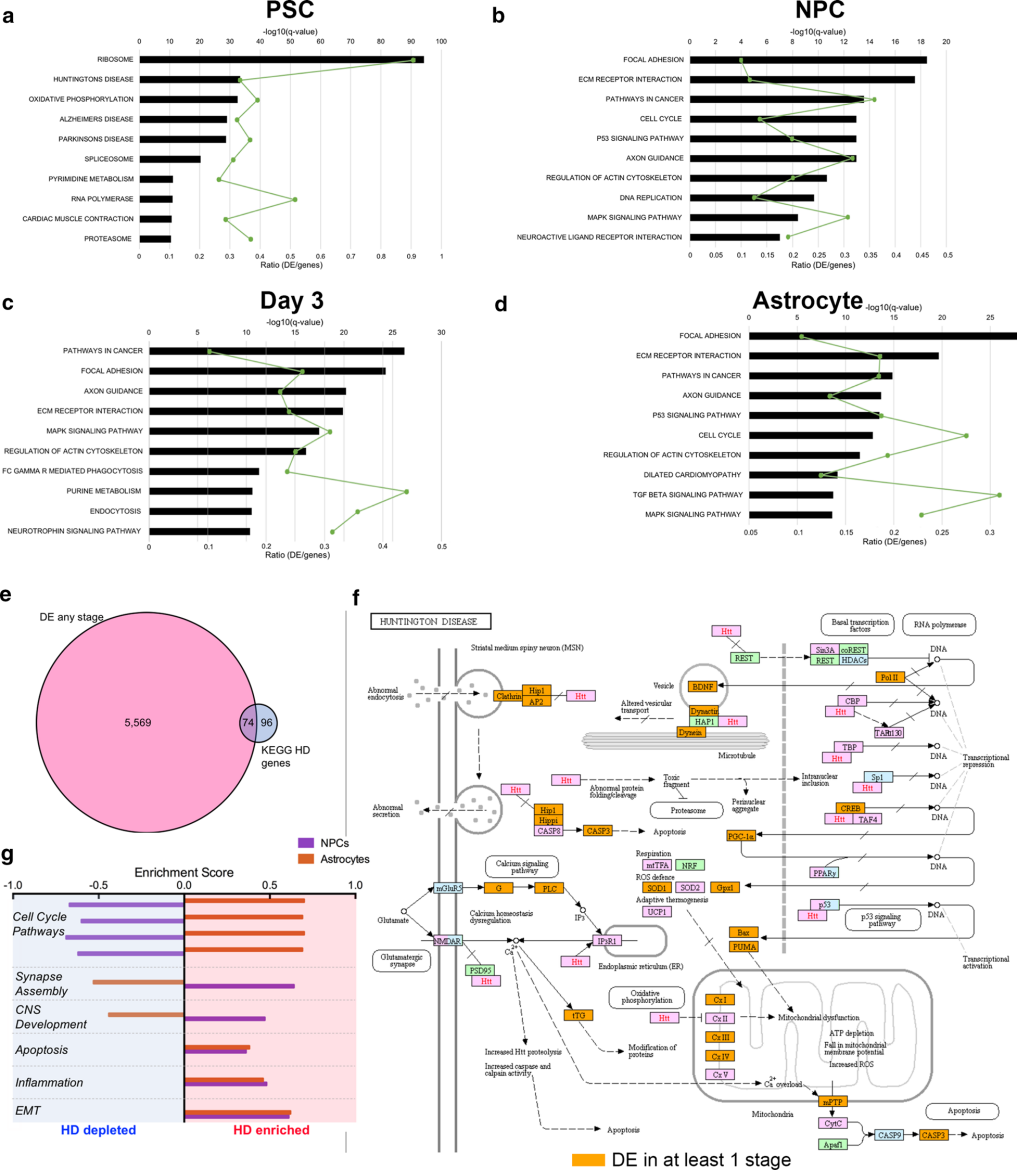
RNA-seq identifies multiple pathways that are altered across HD astrocyte differentiation. Top ten KEGG pathways reported from GO analyses of DE genes at the PSC (**a**), NPC (**b**), day 3 (**c**), and astrocyte (**d**) stages. Pathways are ranked by −log10(*q* value), determined by Benjamini–Hochberg procedures, with threshold set to *q* < 0.05. Line graphs show the ratio of DE genes in each KEGG pathway. **e** DE genes at any stage show overlap with the KEGG Huntington’s Disease pathway. **f** Huntington’s disease pathway showing DE genes (in purple) in HD cells at any stage of astrocyte differentiation. **g** Bar plot depicting the enrichment scores of the top 9 most enriched gene sets, which comprise 6 functional categories. Negative enrichment scores (left; blue) reflect HD-depleted gene sets, and positive enrichment scores (right; red) represent HD-enriched gene sets. NPC GSEA results are shown in purple and astrocyte GSEA results are shown in orange

Gene Ontology (GO) analyses using the Kyoto encyclopedia of genes and genomes (KEGG) pathway gene sets were performed to identify overrepresented pathways across astrocyte differentiation (FDR < 0.05) [99]. Consistent with our previous results, PSCs had the most unique GO results (Fig. 4a), while NPC, day 3 and astrocyte DE genes had similar GO results to one another (Fig. 4b–d; Additional file 1: Figure S5a). Overrepresented KEGG pathways in these 3 stages included DNA replication, p53 signaling pathway, and cell cycle, among others (Fig. 4b–d; Additional file 1: Figure S5a). All of these pathways have been reported to contribute to HD pathogenesis [16, 17, 19, 20, 25, 100–103], but to our knowledge, this is the first study to show that they are altered specifically in astrocytes. Furthermore, 43.5% of the MacaM-annotated genes in the Huntington’s disease *(hsa05016)* pathway are DE at one or more stage of HD astrocyte differentiation (*p* = 5.93e−4; Fig. 4e, f; Additional file 1: Figure S5b). Although the pathway is only significantly overrepresented in the PSC stage (Fig. 4a; Additional file 1: Figure S5b), there are 20 DE genes in NPCs through astrocytes that are in the HD KEGG pathway (11.8%). The relatively low number of annotated HD pathway genes that are DE in astrocytes and their precursors suggests that these cells may have a unique set of pathway alterations in HD compared to other brain cell types such as neurons, as well as PSCs.

Since promoter-proximal THSS enrichment did not correlate strongly with gene expression, we performed GO analysis on the nearest gene to all distal, differential THSSs at each stage, and we obtained similar results to that of the GO analysis of DE genes (Additional file 1: Figure S5c–f; Additional file 6: Table S6), including cell cycle *(hsa04110).* In addition, various pathways involved in signaling and cancer were overrepresented in genes nearest to distal differential THSSs (Additional file 1: Figure S5c–f). Interestingly, GO analysis based on distal differential THSSs in PSCs more closely resembled that of the other 3 stages (Additional file 1: Figure S5c), in contrast to GO results for DE genes at the PSC stage (Fig. 4a).

To specifically identify relevant trends in transcriptional dysregulation during HD astrocyte differentiation, we performed agnostic gene set enrichment analysis (GSEA) [104] using the molecular signature database (MSigDB) [99] at the NPC and astrocyte stages. We found significant enrichment of 9 pathways between HD and WT cells (Fig. 4g) comprising 6 functional categories using this unsupervised approach. Four cell-cycle-related pathways, including E2F target genes, show depletion in HD NPCs and enrichment in HD astrocytes compared to WT cells (Fig. 4g).

Notably, the inverse enrichment trend of E2F target gene expression in HD cells corresponds with the stage-specific trends in E2F motif enrichment that we observed at both promoter-proximal and distal differential THSSs. In addition, gene sets involved in synapse assembly and CNS development are enriched in HD NPCs and depleted in HD astrocytes compared to WT (Fig. 4g). Pathways enriched in HD cells at both stages compared to WT include apoptosis, epithelial-to-mesenchymal transition, and inflammation (Fig. 4g). Given that these pathways have all been previously reported on, we decided to focus on pathways involved in the cell cycle, as they show both overrepresentation [cell cycle (Fig. 4b, d] and the most significant enrichment in the GSEA analysis of our DE genes (Fig. 4g).

### mHTT expression induces progressive upregulation of p53-signaling genes during astrogenesis and in mature astrocytes

mHTT binds to p53, resulting in alteration of its transcriptional activity at select genes and impacting cell-cycle progression, apoptosis, and DNA damage repair [38, 39, 105]; however, the role of p53 signaling in HD is not well understood. Furthermore, p53 signaling and cell-cycle pathways have not been examined in HD astrocytes. Results from our GO analyses of DE genes indicate that significant alterations in p53 signaling occur during HD astrocyte differentiation (Fig. 4b, c) and in mature astrocytes (Fig. 4d). Over half the p53-signaling genes were found to be differentially expressed in at least one stage of astrocyte differentiation (55.1%; *p* = 3.64e−5; Additional file 1: Figure S6a).

**Fig. 5:**
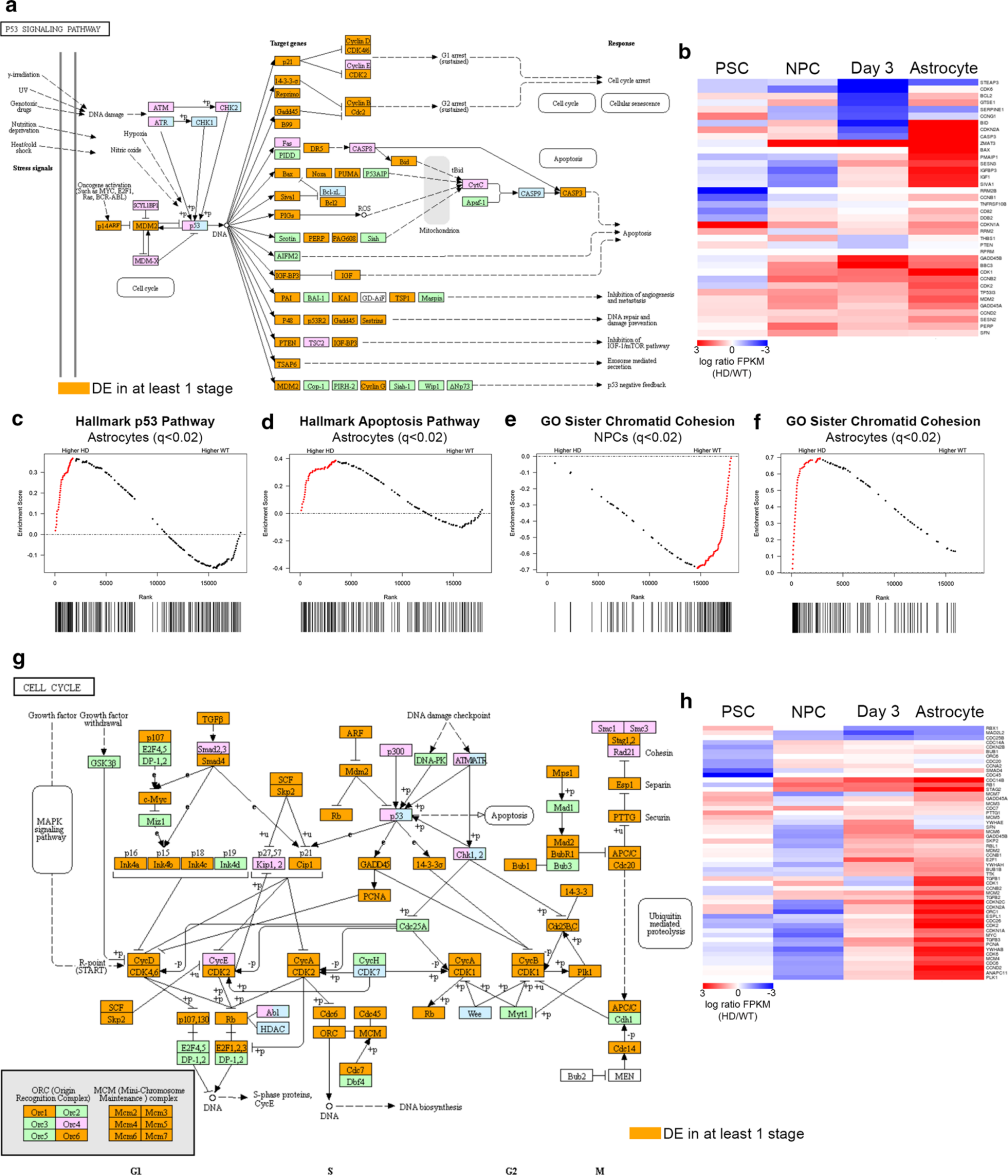
RNA-seq revealed dysregulation of p53 signaling and cell-cycle pathways across HD differentiation. **a** p53 signaling pathway diagram showing DE genes (in purple) in HD cells at any stage of astrocyte differentiation. **b** Heatmap depicting 38 DE genes in the p53 signaling pathway at each stage of differentiation. Red indicates increased expression in HD cells and blue indicates reduced expression in HD cells. Each row corresponds to the same gene and gene names are displayed to the right of the plot. **c**–**f** Cross-sectional GSEA enrichment plots. For GSEA plots, the black lines indicate the position of pathway genes in the expression data rank-sorted between HD and WT samples. Red dots indicate leading edge genes. *q* values are FDR corrected *p* values with alpha = 0.02, or the equivalent. **g** Diagram of the KEGG cell-cycle pathway showing genes DE (purple) in HD cells. **h** Heatmap depicting 54 DE cell-cycle genes in the p53-signaling pathway at each stage of differentiation. Red indicates increased expression in HD cells and blue indicates reduced expression in HD cells. Each row corresponds to the same gene and gene names are displayed to the right of the plot

Widespread transcriptional alterations of the p53-signaling pathway in HD samples (Fig. 5a) included genes involved in cell-cycle arrest (*Cyclin B1*; Additional file 1: Figure S6b), apoptosis (*TP53I3*; Additional file 1: Figure S6c), and DNA repair (*RRM2B*; Additional file 1: Figure S6d). Interestingly, a genome-wide association study (GWAS) found that *RRM2B* contains an SNP, whose minor allele is associated with a 1.6 year reduction in age of onset of HD in humans, implicating *RRM2B* as a potential target of HD therapies [106]. Consistent with this finding, *RRM2B* was significantly upregulated in HD samples at all four developmental stages examined in our study (Additional file 1: Figure S6d).

Although no changes in expression of the *p53* gene itself were observed, our data indicate that a large portion of the p53-signaling pathway was DE (Fig. 5a; Additional file 1: Figure S6a). Consistent with this, there is evidence that altered p53 function can be posttranslational, with upregulation at the protein level, but not in mRNA in HD [38]. Furthermore, important upstream effectors of p53 signaling, *p14ARF* (also known as *CDKN2*; Additional file 1: Figure S6e) and *MDM2* (Additional file 1: Figure S6f), are upregulated in NPCs, day 3, and astrocytes. A heatmap of the 38 DE genes in the p53 pathway demonstrates progressive upregulation of p53 pathway genes in NPC, day 3 and astrocyte samples (Fig. 5b; Additional file 1: Figure S6c–f). In support of this, GSEA using Hallmark gene sets shows significant enrichment of *p53-*signaling genes in HD astrocytes (Fig. 5c), but not HD NPCs (Additional file 1: Figure S6g), compared to WT cells. In addition, cross-sectional GSEA results show the expression of genes in the apoptosis pathway is enriched in HD astrocytes (Fig. 5d), but not NPCs (Additional file 1: Figure S6h). Furthermore, agnostic longitudinal GSEA, comparing NPC to astrocyte gene expression in HD and WT separately, reports a significant enrichment of apoptosis pathway genes in HD astrocytes but not WT astrocytes (Additional file 1: Figure S6i, j). Taken together, our results suggest that elements of the p53-signaling pathway are altered due to mHTT expression at all stages of astrocyte differentiation and involve the progressively altered expression of genes related to the cell-cycle, apoptosis, and DNA repair.

### mHTT expression results in altered cell-cycle pathway gene expression during astrocyte differentiation

p53 upregulation has been associated with cell-cycle deficits observed in HD models; however, the global downstream transcriptional consequences of this remain unknown [105, 107]. Our GO analyses identified alterations of the cell-cycle pathway in HD cells across differentiation (Fig. 4b, d; Additional file 1: Figure S5a). Further unsupervised GSEA revealed significant, temporally dependent HD enrichment of multiple pathways involved in the cell cycle across astrocyte differentiation (Fig. 4g). Cross-sectional enrichment analyses demonstrate significant depletion in the expression of genes in sister chromatid cohesion (Fig. 5e), *G2* *M* Checkpoint (Additional file 1: Figure S7a), and sister chromatid segregation (Additional file 1: Figure S7c) pathways in HD NPCs compared to WT. Alternatively, HD astrocytes display significant enrichment of these pathways compared to WT cells (Fig. 5f; Additional file 1: Figure S7b, d). This inverse enrichment across differentiation is illustrated by DE of genes such as *Cyclin B1* (Additional file 1: Figure S6d) and *CDK1* (Additional file 1: Figure S7f), which regulate checkpoints during the cell cycle.

Altered expression is observed throughout the cell-cycle pathway (Fig. 5g), with nearly half the gene set (43.5%; *p* = 3.30e−3; Additional file 1: Figure S7e) showing DE in at least one stage of astrocyte differentiation. While overall upregulation of cell-cycle gene expression is observed between NPC and astrocyte stages in HD samples, as seen with *Cyclin D1* (Additional file 1: Figure S7g), a small number of DE genes, such as *Cyclin D2* (Additional file 1: Figure S7h), show reduced expression compared to WT cells across differentiation. Figure 5g provides insight into potential downstream cell-cycle consequences of altered p53 signaling in HD. A heatmap showing DE of cell-cycle pathway genes across all four stages of differentiation illustrates the inverse expression profiles of HD NPCs and HD astrocytes compared to respective WT cells (Fig. 5h).

Our data suggest that alterations of the p53 signaling and cell-cycle pathways in HD occur early in neural development and are progressive, with HD astrocytes showing increased expression of a majority of pathway members compared to WT astrocytes (Fig. 5d, H; Additional file 1: Figure S6d–h). As p53 signaling is responsible for the regulation of multiple pathways, these analyses cannot directly conclude that p53 signaling is responsible for the altered cell-cycle transcription profile observed. However, as many DE genes in the p53 pathway are also in the cell-cycle pathway and both pathways show similar expression trends (Fig. 5d, h), we continued with investigations of HD-mediated cell-cycle aberrations during astrocyte differentiation.

### mHTT expression alters E2F target genes during astrocyte differentiation

**Fig. 6:**
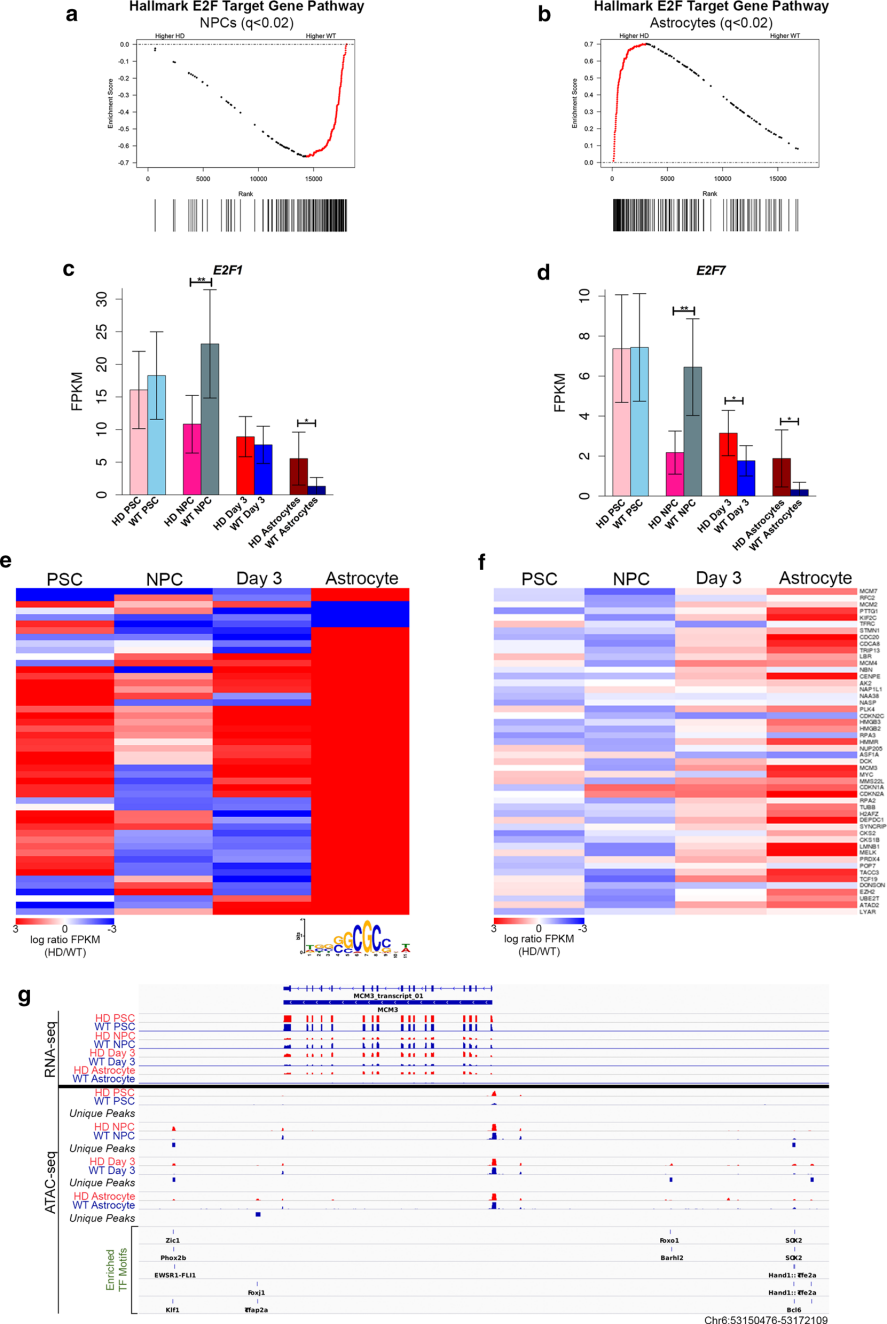
Aberrant E2F regulation coincides with increased p53 signaling and cell-cycle gene expression in HD astrocytes. Cross-sectional GSEA enrichment plots shows significant depletion of E2F target gene expression in HD NPCs (**a**) and significant enrichment in HD astrocytes (**b**) compared to WT cells. Black lines indicate E2F target gene positions in rank-sorted expression data between HD and WT samples. Red dots indicate leading edge genes. *q* values are FDR corrected *p* values with alpha = 0.02, or the equivalent. RNA-seq expression data for *E2F1* (**c**) and *E2F7* (**d**) showing differential expression corresponding with motif accessibility. Average FPKM for each sample was plotted. Error bars show 95% confidence intervals (**p < 0.001 and *p < 0.01, differential expression analysis). **e** Heatmap depicting nearest differential ATAC-seq peaks to E2F target genes that are DE in at least one stage of astrocyte differentiation. Both proximal and distal peaks are included. Nearest differential ATAC-seq peaks are arranged according to hierarchical clustering, and correspond to the gene order in panel F. The E2F1 motif is shown below the heatmap. **f** Heatmap depicting differential expression of E2F target genes across differentiation. Each row corresponds to the same gene and gene names are displayed to the right of the plot. For both heat maps, red represents HD enrichment and blue indicates HD depletion. **g** Track view of RNA-seq and ATAC-seq data, as well as motifs present in differential peaks, at *MCM3*, an example E2F target gene. HD signal is shown in red and WT in blue. Significant differential peaks are indicated in the tracks below ATAC-seq tracks at each stage. TF motifs enriched in differential peaks are displayed at the bottom

Unsupervised GSEA analysis comparing HD and WT cells at the NPC or astrocyte stage consistently reports that E2F target genes are the most significantly enriched gene set between our samples (Fig. 6a, b). As with the other cell-cycle pathways described, inverse enrichment shows significant depletions in E2F target gene expression in HD NPCs compared to WT NPCs (Fig. 6a), while HD astrocytes show significant enrichment compared to WT (Fig. 6b). Furthermore, longitudinal GSEA of WT and HD samples demonstrates that in WT cells, E2F target gene expression is enriched at the NPC stage compared to the astrocytes stage (*q* < 0.02; Additional file 1: Figure S8b). However, HD cells show no significant enrichment of E2F target genes across differentiation (Additional file 1: Figure S8a), demonstrating that altered regulation of these genes begins at the NPC stage and persists through HD astrocyte differentiation.

Interestingly, motif analysis of differential promoter-proximal THSSs reported E2F1, E2F2, E2F7, and E2F8 motifs show corresponding trends with GSEA findings; at the NPC and astrocyte stage: NPCs show WT enrichment of E2F motifs at accessible promoters, while astrocytes show HD enrichment of the E2F1 motif (Fig. 1f, g). The stage-specific differential expression of *E2F1* (Fig. 6c), which corresponds to promoter motif enrichment and expression profiles of target genes, prompted us to examine the expression of all E2F family members across astrocyte differentiation (Fig. 6c, d; Additional file 1: Figure S8c–h). While only *E2F1* and *E2F7* demonstrate differential expression patterns corresponding to GSEA results (Fig. 6c, d), most other E2F TFs are DE in at least one stage of astrocyte differentiation (Additional file 1: Figure S8c–h).

To better characterize the interplay between altered chromatin accessibility and E2F target gene expression, we first examined promoter-proximal ATAC-seq enrichment at genes differentially expressed in at least one stage, and consistent with our genome-wide results, we found that differences in promoter accessibility did not correlate strongly with differential expression of E2F target genes across HD differentiation (Additional file 1: Figure S9a, b). Overall, it appears that alterations in E2F target promoter accessibility lag behind changes in expression, suggesting that other regulatory mechanisms may drive differential expression of E2F target genes in HD astrocytes.

This prompted us to examine nearest differential THSSs to E2F target gene promoters (Fig. 6e), regardless of whether they are proximal or distal to the promoter. We observe a dramatic increase in THSS accessibility in HD astrocytes, which more closely reflect expression profiles for corresponding E2F target genes (Fig. 6e, f). Similarly, the overall HD depletion of the nearest differential THSS at the NPC stage more closely resembles the pattern of differential expression in the corresponding genes at that stage (Fig. 6e, f). It is not surprising that these correlations are strong, but not perfect, since differential THSS proximity does not guarantee that the associated target gene is interacting with or regulated by these nearest differential THSSs. In PSCs and day 3 cells, where there is relatively less differential expression of the E2F target genes, this correlation is weaker, suggesting that when an E2F target gene is significantly DE, it may tend to interact with or be regulated by its nearest differential THSS. At all stages, the nearest differential THSSs occurred within 250 kb of E2F promoters in most cases (Additional file 1: Figure S9c).

Motif enrichment analyses of the nearest differential THSS-to-E2F target genes in NPCs and astrocytes was performed and revealed the presence of binding sequences of a large number of different TFs (Additional file 7: Table S7). Strikingly, there is no single TF whose motif is found at the majority of the differential THSSs: any one reported TF motif enrichment only occurs at a few differential THSSs in each stage, indicating that each E2F target gene may be regulated by a distinct set of trans-acting TFs in this case.

An example of a differentially expressed E2F target gene, *MCM3*, is shown in Additional file 1: Figure S9d. *MCM3* is DE in NPC, day 3 and astrocyte samples, but no differential promoter-proximal accessibility is observed in its promoter. However, corresponding differential THSS enrichment is observed both upstream of the promoter (around 15 kb and 30 kb), as well as up to 10 kb downstream of the transcription termination site (Fig. 6g). Notably, *MCM3* is a well-established marker of proliferation and is one of six mini-chromosome maintenance (MCM) genes targeted by E2F TFs within the cell-cycle pathway. All 6 E2F-regulated MCM genes show DE in at least one stage of astrocyte differentiation (Fig. 5g; Additional file 1: Figure S9d-i).

**Fig. 7:**
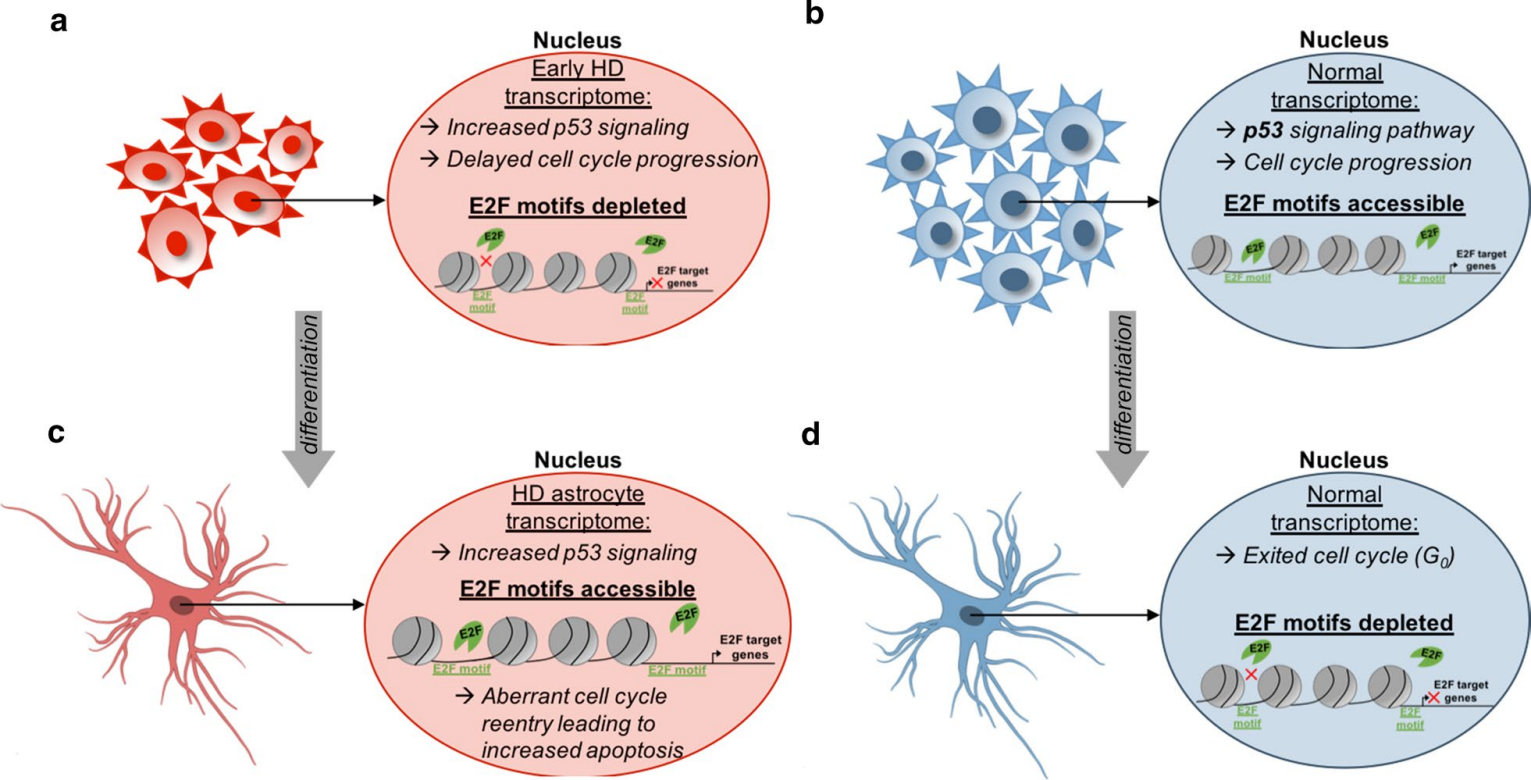
Schematic model showing cell cycle, p53 signaling, and E2F target gene regulation across HD astrocyte differentiation. **a** HD NPCs show increased expression of p53 signaling genes, decreased expression of cell cycle and E2F target genes, which coincides with depleted promoter-proximal and distal accessibility of the E2F TF motif, while WT NPCs (**b**) show normal cell-cycle progression and accessible E2F TF motifs genome-wide. **c** HD astrocytes show upregulation of p53 signaling, apoptosis, cell cycle and E2F target gene expression, along with increased E2F TF motif accessibility, suggesting cell-cycle re-entry leading to apoptosis. **d** In comparison, WT astrocytes show depleted accessibility of E2F TF motifs, and have low expression of p53 signaling, E2F target and cell-cycle genes, indicating they are quiescent

In addition to cell-cycle and apoptosis-specific genes, E2F TFs also regulate several epigenetic factors that demonstrated differential expression during astrocyte differentiation. For example, the H3K27 methyltransferase, *EZH2,* is downregulated in HD NPCs, but is overexpressed in HD astrocytes, following the expression pattern of cell cycle and apoptosis-related pathway genes across differentiation (Additional file 1: Figure S9j). *DNMT1* (Additional file 1: Figure S9k) and *UHRF1* (Additional file 1: Figure S9l) also show depleted expression in HD NPCs, but by day 3 of astrocyte differentiation onward, these regulators of DNA methylation are significantly over expressed compared to WT cells. In addition, *HMGN3* displayed differential expression in NPCs and astrocytes (Additional file 1: Figure S9m); however, in contrast to the inverse differential expression of most other E2F target and cell-cycle genes, *HMGN3* is significantly overexpressed in HD NPCs and depleted in HD astrocytes. HMGN3 binds to and remodels accessibility of chromatin to directly modulate transcription [108]. Most interestingly, HMGN3 has been suggested to be important for normal astrocyte function [108, 109]. Thus, the observed *HMGN3* upregulation in HD NPCs and depletion in HD astrocytes (Additional file 1: Figure S9m) is consistent with our previous findings of other astrocyte-specific genes during HD differentiation (Additional file 1: Figure S2c, d) and provides evidence that premature initiation and incomplete terminal differentiation occurs during HD astrogenesis. In summary, we observe dysregulation of E2F target gene expression that not only coincides with alterations in cell-cycle progression and apoptosis pathways, but also reports aberrant expression of chromatin remodeling and epigenetic proteins, possibly contributing to incomplete astrocyte differentiation in HD cells (Fig. 7a–d).

## Discussion

Here, we analyzed transcription and chromatin accessibility dynamics during astrocyte differentiation in WT and transgenic HD Rhesus macaque PSCs using an unbiased, integrative approach. We identified genome-wide alterations in gene expression across differentiation; however, persisting DE patterns were only evident from the NPC to astrocyte stage. PSCs display a more unique DE profile. DE genes identified by RNA-seq in HD Rhesus macaque cells show considerable overlap with RNA-seq data sets from in vitro and in vivo human HD models [17, 25] and replicated numerous findings from other non-glial HD models [16, 19, 20, 100–103], providing further evidence that our HD Rhesus macaque model recapitulates HD-associated phenotypes and may offer a reliable and translatable system for investigation of neurodegenerative mechanisms and identification of potential effective therapeutic strategies.

Furthermore, we found extensive alterations in THSS enrichment and thus TF occupancy during HD astrocyte differentiation. Overall, HD cells show depletion of promoter-proximal accessibility; however, most differential THSSs are found at enhancers and other distal regions and are more strongly correlated to changes in gene expression than promoter accessibility. Differential distal THSSs display a distinct and progressive signature of dysregulation as HD NPCs differentiate into astrocytes. Interestingly, the NPC and day 3 stages each have more than double the number of differential THSSs than either the PSC or astrocyte stage. We also identified several TFs that show stage-specific motif enrichment in differential THSSs along with corresponding changes in expression, often in subsequent stages, such as RFX2, RFX4, and FOSL2. To our knowledge, these TFs have not been previously implicated in HD, thus warranting further investigation. Our data suggest that aberrant priming of epigenomic profiles occurs in HD NPCs, after committing to a neural lineage, and leads to compounding deregulation of chromatin accessibility, TF binding and transcription during HD astrocyte development. As enhancer activity is known to be highly context specific and altered by disease pathology, it is important to point out that H3K27ac, which was used to define PABEs in the present study, has been shown to be altered in HD [30, 42, 43, 47, 49, 51]. While our results offer the first evidence of altered chromatin accessibility across HD astrocyte differentiation, further investigations of chromatin signatures and TF binding at regulatory elements across differentiation and in various neural cell types are necessary to confirm our findings and to fully understand the interplay of epigenome and transcriptome dysregulation in HD.

PSCs show the most distinct differential expression and THSS profiles, while progressive trends were observed from HD NPCs to astrocytes. Based on the clustering patterns observed in both the RNA-seq and ATAC-seq data, we hypothesize that, broadly speaking, there are two distinct stages of HD-mediated dysregulation that occur during astrocyte development: one in PSCs and the other beginning in the NPC stage, after commitment to a neural lineage. While there are differences in HD-mediated alterations between NPCs and astrocytes, these differences tend to involve similar pathways, whereas HD-mediated alterations in PSCs involve completely different pathways. This hypothesis is consistent with findings from a study profiling H3K27me3 and H3K4me3 dynamics during early HD differentiation, which demonstrated that HD ESCs and NPCs display distinct chromatin signatures, suggesting that differential HD-mediated dysregulation occurs across development [44]. This study did not investigate any further stages of neural differentiation, but RNA-seq findings have widely reported alterations of pathways associated with neurodevelopment in HD [16, 17, 19–21, 25], indicating that early alterations may persist across development. It is possible that this dichotomy between PSCs, and NPCs and their derivatives results from the well-documented impact of mHTT on neurodevelopment specifically [23, 110, 111]; as PSCs transition to NPCs and commit to a neural lineage, neurodevelopmental programs are initiated and, in the presence of mHTT, are progressively dysregulated.

RNA-seq data revealed alterations of pathways previously implicated in HD, but not specifically in astrocytes, and implicate dysregulation of multiple cell-cycle pathways, including p53 signaling, during HD astrogenesis. p53 acts as a TF to regulate many cellular processes and has been implicated in cell-cycle dysregulation in HD [105, 107]. We observed a significant, progressive upregulation of p53-signaling genes in HD between the NPC and astrocyte stages that coincide with enrichment in cell cycle and apoptosis pathways. Notably, upstream regulators of p53 signaling, *CDKN2A* and *MDM2*, were upregulated in HD day 3 and astrocytes. In addition, the p53 pathway gene *RRM2B*, which plays a role in DNA synthesis and repair [112], was upregulated in HD cells at all stages of astrocyte differentiation. Interestingly, a genome-wide association study (GWAS) identified the minor allele of *RRM2B* as a genetic modifier associated with accelerated onset in HD patients [113]. Given this, we suggest *RRM2B* expression in astrocytes as a potential candidate for follow-up studies of healthy astrocyte-mediated HD attenuation. Based on our findings, we hypothesize that observed alterations in p53 signaling contribute, potentially via multiple mechanisms, to the progressive alteration of the cell cycle and HD pathogenesis during astrocyte differentiation. Our model and others may be useful in confirming this hypothesis and potentially leading to an understanding of how to attenuate HD pathology in therapeutic treatments.

Stage-specific alterations in the cell cycle have been identified in mouse and human models of HD [17, 18, 20, 100, 105, 107, 114, 115]. For example, mHTT has been shown to disrupt spindle orientation and thus mitotic division [18]. Further molecular evidence from 293 cells expressing mHTT has demonstrated that the appearance of cell-cycle arrest and mitotic defects coincides with an observed increase in p53 expression [105]. Consistent with this, we observe that changes in cell-cycle gene expression follow the same trends as those observed in the p53 pathway, with depletion in HD NPCs and enrichment in HD astrocytes. Consistent with this, we identified four specialized cell-cycle-associated pathways that showed substantial, significant differential enrichment between HD and WT in NPCs and astrocytes: *E2F* target genes, G2/M checkpoint, sister chromatid cohesion, and sister chromatid segregation.

Of particular interest is the E2F target gene set. E2F TFs are most widely known for their role in cell-cycle regulation; with E2F1, E2F2, and E2F3 serving as activators, while E2F7 and E2F8 are repressors [116]. E2F1 is a TF that regulates the G1/S checkpoint of the cell cycle and has been shown to regulate cell death through a p53-dependent manner [117–119], and also plays a role in other cellular processes, such as autophagy [120]. Recent evidence suggests that E2F1 is also capable of initiating apoptosis by directly inducing the expression of *CDKN2A*, which forms a complex with *MDM2* and p53 to initiate cell-cycle arrest and apoptosis [121, 122]. In addition, aberrant expression of E2F TFs can cause cell-cycle re-entry and apoptosis [123, 124]. Consistent with these findings, we observe DE of most E2F TFs and upregulation of target gene *CDKN2A*, along with increased p53 signaling, cell cycle, and apoptosis pathway activation in HD astrocytes, suggesting that a similar mechanism is occurring in our HD cells during differentiation. Although the roles of individual E2F TFs in p53 signaling and cell-cycle dysregulation in HD are not well characterized, E2F1 dysregulation has also been implicated in neuronal death in neurodegenerative diseases such as Alzheimer’s disease [125] and Parkinson’s disease [126, 127], and was reported to be upregulated in human HD brains [115]. Taken together, our results support the hypothesis that aberrant cell-cycle re-entry during HD astrocyte differentiation induces apoptosis via an E2F1-p53-dependent mechanisms. Future molecular studies are necessary to characterize these events in HD cells.

The present study shows global alterations in E2F motif enrichment at differential THSSs across astrocyte differentiation that coincide with E2F target gene expression profiles. We found that the nearest differential THSS-to-E2F target promoters more strongly associated with gene expression than promoter accessibility. Interestingly, DE profiles of E2F target genes precede changes in nearby, differential THSS enrichment, suggesting that other regulatory mechanisms must contribute to E2F dysregulation in HD astrocytes. We also provide evidence that the altered expression profiles of E2F target genes during HD astrocyte differentiation may be regulated by differential binding of multiple different TFs at distal regulatory regions. However, future studies are necessary to shed light onto the regulatory interactions underlying the DE of E2F target genes.

Interestingly, among the E2F target genes that were found to be DE during differentiation were several epigenetic factors, including regulators of histone and DNA methylation (*EZH2, DNMT1, UHRF1*) and chromatin remodelers (*HMGN3*). All three methyltransferases showed depletion in HD NPCs and upregulation in HD astrocytes. EZH2 is part of the Polycomb repressive complex 2 (PRC2), which is critical for ESC differentiation into NPCs [36, 128, 129]. Although HTT is known to directly interact with EZH2, the influence of mHTT on EZH2-mediated H3K27me3 is not well defined [36, 44]. In addition, global alterations in DNA methylation in HD have been reported and were interestingly found to be differential across brain regions [130, 131], highlighting the need for extensive characterization of the HD epigenome both across neural cell types and throughout neurodevelopment. Both *DNMT1* and *UHRF1* activities are associated with the cell cycle; DNMT1-mediated DNA methylation is bound by UHRF1, which also binds histone H3 lysine 9 trimethylation (H3K9me3) to orchestrate higher, multi-layer epigenetic regulation of transcription. Of note, H3K9me3 has been reported to be enriched in HD human and mice brains [48, 132]. Furthermore, it has been demonstrated that inhibiting DNMTs provides a neuroprotective effect in HD mice [133]. Given their association with less accessible chromatin and our findings that HD promoters show depleted accessibility, while distal accessibility is largely HD enriched across differentiation, it would be interesting to more closely examine the consequences of altered *DNMT1* and *UHRF1* expression on DNA methylation and H3K9me3 dynamics during HD astrocyte differentiation.

Finally, in contrast to DE profiles for other E2F target genes, *HMGN3* expression is significantly increased in HD NPCs, but is significantly depleted in HD astrocytes. This chromatin remodeling enzyme has been shown to control astrocyte differentiation from NPCs [134] and is important to astrocyte function [108, 109]. The present study provides evidence that multiple astrocyte-specific genes, such as *HMGN3, GFAP, APOE*, and *LCN2*, are significantly upregulated early in HD differentiation (NPC and day 3 stages) both compared to corresponding WT samples and to expression levels observed in HD astrocytes. In support of previously reported evidence from heterogenous neural populations, this finding suggests that our HD cells prematurely upregulated astrocyte differentiation pathways, but never fully develop mature astrocyte transcription profiles [25], causing early and progressive impairments that not only impact astrocytes themselves, but may also leave their associated neuron population more vulnerable to environmental stressors and neurotoxicity [70].

Alteration of E2F TFs and cell-cycle pathways provides further support for this, as E2F TFs are also known to regulate the switch between proliferating NPCs and differentiation expression profiles that require exit from the cell cycle for commitment to a specific neural lineage, such as glial cell [116, 135, 136]. In fact, E2F4 and E2F5 regulate differentiation of NPCs into specific neural lineages [116], along with *E2F1* downregulation [135, 136]. There is also evidence that each E2F TF regulates a distinct gene set in a stage-specific manner across neurodevelopment [116, 123, 124]. Consistent with this, WT NPCs show increased proliferation and enrichment of cell-cycle pathways, specifically E2F target genes. Once induced to differentiate into astrocytes, WT cells show dramatic down regulation of cell cycle and E2F target genes by day 3, which persists in WT astrocytes. HD NPCs display downregulation of cell cycle and E2F target genes and delayed proliferation compared to WT cells. Upon differentiation into astrocytes, E2F TF expression is upregulated in HD cells with increased cell-cycle gene expression and aberrant cell-cycle re-entry that coincides with enrichment of p53 signaling and apoptosis pathways. This demonstrates that HD Rhesus macaque cells are unable to properly regulate E2F TFs and switch to the transcriptome required for the generation of mature astrocytes. This incomplete activation of astrocyte markers in HD cells may be a downstream consequence of early E2F dysregulation. Future studies should focus on identifying the initial mechanisms of E2F dysregulation and the direct downstream transcriptional consequences of aberrant E2F activity during HD astrocyte differentiation. Taken together, these results indicate that E2F TF dysregulation during astrocyte differentiation has consequences that extend beyond cell-cycle regulation.

## Conclusions

We observe E2F deregulation that not only impacts cell-cycle progression and apoptosis pathways, but also alters the expression of chromatin remodeling and epigenetic proteins, possibly resulting in suboptimal astrocyte differentiation in HD cells. Furthermore, our study has provided new genetic and epigenetic insights into the effects of mHTT expression on astrocytes and has provided evidence for numerous hypotheses on the role of astrocytes and their precursors in the progression of HD in the presence of mHTT. Our HD model may be useful in directly testing these hypotheses and others aimed at further characterizing the role of astrocytes and their precursors in HD, and in identifying therapies involving astrocytes that can attenuate HD.

## Methods

### Rhesus macaque PSC cultures

WT Rhesus ESCs and transgenic Rhesus HD iPSCs were previously established [90]. Transgenic HD iPSCs expressed both exon 1 of the human *HTT* gene with 65 CAG repeats and GFP under the control of the human polyubiquitin-C (*UBC*) promoter [88, 90]. Rhesus PSCs were cultured on mouse fetal fibroblast (MFF) feeder cells in ESC culture media [Knockout-Dulbecco’s modified Eagle’s medium (KO-DMEM; Invitrogen) with 20% Knockout Serum Replacement (KSR; Invitrogen), 1 mM glutamine, 1 nonessential amino acids (NEAA), and 4 ng/mL of human basic fibroblast growth factor (bFGF; Chemicon, Inc., Tumecula, CA)]. In addition, Rhesus PSC cultures were expanded by mechanical passaging. Since these Rhesus PSC lines were previously used to derive stable NPC cultures [89, 137, 138], PSCs were collected for experimental analysis, but not further differentiated into NPCs in this study.

### Rhesus macaque NPC culture maintenance

NPCs were maintained and expanded as previously described [89, 137, 138]. Briefly, P/L-coated [20 µg/mL poly-l-ornithine (Sigma) and 1 µg/cm2 laminin (Sigma)] cell culture dishes were used to culture cells in neural proliferation medium [Neurobasal-A medium (Life Technologies) with 1 × penicillin/streptomycin (Invitrogen), 2 mM of l-glutamine, 1× B27 (Life Technologies), 20 µg/mL of bFGF (R&D), and 10 ng/mL of mLIF (Millipore)]. Cells were maintained at 37 °C and 5% CO2 and media was changed every 2 days. Upon reaching confluence, cells were passed at a 1:1.5 ratio.

### In Vitro Astrocyte Differentiation

The astrocyte differentiation protocol used in this study was based on a previously published protocol [92]. For in vitro differentiation of NPCs into astrocytes, Rhesus macaque NPCs were seeded with the seeding density of 2x10^5^ cells/cm^2^ on P/L-coated culture plates. Neural proliferation media were replaced with astrocyte differentiation media [Neurobasal-A medium (Life Technologies) with 1x penicillin/streptomycin (Invitrogen), 2 mM of l-glutamine, 500 nM of azacytidine (Aza-C; Sigma), 20 nM of trichostatin (TSA; Sigma), 20 ng/mL of bone morphogenetic protein 2 (BMP2; R&D), and 1x B27 (Life Technologies)]. After 2 days, the Aza-C and TSA were removed, and cells were cultured in the astrocyte differentiation media for an additional 28 days.

### Quantitative reverse transcription PCR (RT-qPCR)

Total RNA was prepared from cell samples using TRIzol^®^ (Life Technologies), followed by DNA digestion using Turbo DNA-free TM kit (Invitrogen) according to the manufacturer’s instructions. RNA samples (500 ng) were used to synthesize cDNA using a High-Capacity cDNA Reverse Transcription Kit (Applied Biosystems). RT-qPCR was performed on CFX96 Real-Time Detection System (Bio-Rad) using either IQTM SYBR^®^ Green Supermix (Bio-Rad) or TaqMan™ Gene Expression Master Mix (Applied Biosystems) depending on the primers used. RT-qPCR primer sequences are listed in Additional file 1: Table S1. PCR conditions for SYBR Green primers: Initial 95 °C activation step for 30 s followed by amplification cycles 95 °C for 10 s and 55 °C for 30 s for 50 cycles. Reaction conditions for TaqManTM gene expression primers: initial 95 °C activation step for 10 min followed by amplification cycles 95 °C for 15 s and 60 °C for 60 s for 40 cycles. Unless otherwise mentioned, one-way analysis of variance (ANOVA) was used for statistical comparison of RT-qPCR data. Statistical tests were performed using SPSS 23 (IBM) and graphs were prepared using GraphPad Prism 6 (GaphPad Software, Inc.). Bar graphs reflect the mean ± standard error of the mean (SEM) values for each sample. Statistical significance was established *p* < 0.05.

### RNA-seq experiments

Cell cultures were harvested and counted. Five hundred thousand cells were pelleted and homogenized in 350 µL TRIzol^®^ (Life Technologies). Cell homogenates were briefly vortexed and stored at − 80 °C. The RNA was extracted using Qiagen miRNeasy Mini Kit with DNase digestion. The RNA quantity and quality were validated using Nanodrop 2000 Spectrophotometer and Agilent’s 4200 Bioanalyzer Capillary electrophoresis. Total RNA (10 ng) was used as an input for mRNA amplifications using Clontech Smarter V4 chemistry according to manufacturer’s instructions. Amplified mRNA was fragmented, and barcodes were added using Illumina’s Nextera XT kits. Amplified Libraries were validated by Agilent 4200 Tapestation and quantified using a Qubit fluorimeter. Libraries were normalized, pooled, and clustered on an Illumina HiSeq 3000/4000 Flowcell using the Illumina cBOT. The libraries were sequenced on an Illumina HiSeq 3000 system in 101-base single-read reactions with multiplexing to achieve approximately 20 million reads per sample. RNA-seq experiments were performed in three different replicates.

### Assay for transposase-accessible chromatin using sequencing (ATAC-seq)

ATAC-seq was performed using the Omni-ATAC protocol [94]. Cell cultures were harvested, washed in cold 1x PBS, and counted. One hundred thousand cells were resuspended in 50 µL cold resuspension buffer (RSB; 10 mM Tris–HCl pH 7.4, 10 mM NaCl, and 3 mM MgCl_2_) containing 0.1% NP-40 (Sigma), 0.1% Tween-20 (Sigma), and 0.01% digitonin (Abcam ab141501) and incubated for 3 min on ice. Following lysis, 1 mL RSB with 0.01% Tween-20 was added to the samples. Samples were centrifuged for 10 min at 4 °C/500 x g and the supernatant was carefully removed. Nuclei were then resuspended in the transposase reaction mix (25 µL 2x TD buffer, 2.5 µL Tn5 transposase, 0.5 µL 10% Tween-20, 2.5 µL 1% digitonin [0.05% final concentration], and 19.5 µL water) and incubated at 37 °C for 30 min in a thermomixer with shaking at 600 r.p.m. Following the reaction, samples were treated with Proteinase K (Fisher 25530015) at 55 °C for 2 h and genomic DNA was isolated via phenol:chloroform:isoamyl alcohol extraction and ethanol precipitation. Library preparation was performed using 2x KAPA HiFi mix (KAPA BIOSYSTEMS INC #kk4604) and 1 mM indexed primers under the following PCR conditions: 72 °C for 5 min; 98 °C for 30 s; and 8–12 cycles at 98 °C for 10 s, 63 °C for 30 s, and 72 °C for 1 min. Libraries were sequenced using Illumina HiSeq 2500. ATAC-seq experiments were performed on two replicates.

### Analysis of RNA-seq and ATAC-seq data

RNA-seq and ATAC-seq data were analyzed as follows for all subsequent analysis except the ANOVA. All data were aligned to “MacaM_Rhesus_Genome_Annotation_v7.8.2″ [139]. RNA-seq data were aligned using Tophat2 v2.1.0 [140] with the flags, “–no-mixed –no-discordant”, and differentially expressed genes were called using cuffdiff v2.1.1 [93] using default parameters and a cutoff of *q* < 0.01. ATAC-seq reads were first trimmed using pyadapter_trim.py and then aligned using bowtie version 2 2.2.6 [141] with the flag, “-X 2000”, then duplicates were removed from ATAC-seq samples using picard-tools-2.1.7 MarkDuplicates (http://broadinstitute.github.io/picard/). To adjust for fragment size, we aligned all reads as + strands offset by +4 bp and—strands offset by − 5 bp [142]. Non-nucleosomal ATAC-seq fragments (length < 125 bp), as well as mononucleosome fragments (between 171 and 254 bp), were then isolated for downstream analysis of TF binding.

Peaks of non-nucleosomal ATAC-seq reads were called on each replicate of each sample separately using macs2 version 2.1.0.20151222 [143] in BAMPE mode with default parameters. Peaks from separate replicates were then merged using bedtools merge [144] to get a single set of THSSs (Tn5 hypersensitive sites) for each sample. These were then input into MAnorm3 [145] to call differential peaks (HD vs. WT) at each stage. We required that differential peaks had a logCPM (Counts per Million), averaged over HD and WT samples, > 1, as reported by MAnorm3. In addition, we required that they either had *q* < 0.01 or a logFC (HD/WT) magnitude > 3 and *p* < 0.01. Differential peaks occurring on chr2a and chr2b were excluded from downstream analysis. MAnorm3 was also used to call differential nucleosome occupancy at promotors, by taking a 2 kb region centered at TSSs. *p* values for the overlap of DE genes with published lists and KEGG pathways were calculated using Fisher’s exact test based on a 2 × 2 contingency matrix:

$$ \begin{array}{*{20}c} {\text{N11}} & {\text{N12}} \\ {\text{N21}} & {\text{N22,}} \\ \end{array} $$

where N11 = # of DE genes in the given set; N12 = # of genes in the given set that are not DE; N21 = # of DE genes in the entire set of annotated genes considered; N21 = # of non-DE genes in the entire set of annotated genes considered.

### Differential heatmaps

To generate the differential heatmaps presented in this paper, fragments per kilobase per million mapped reads (FPKM) values were calculated in each sample separately using pooled replicates. Then, log ratios were calculated as log((FKM_HD + 0.0001)/(FKM_WT + 0.0001)) to avoid division by zero errors. In cases, where FPKM_HD < 1 and FPKM_WT < 1, the value of the log ratio was set equal to 0. The base of the log for these calculations was 2.

### Anova

Reads were aligned to the Rhesus macaque (Macaca mulatta) assembly (MacaM_Rhesus_Genome_v7.fasta) [139] using STAR software (v2.5.2b) [146]. Transcripts were annotated using the UNMC rhesus annotation v 7.6.8 and unsorted bam files were sorted and indexed using samtools and converted to HTSeq-count format. Estimates of genewise and isoformwise expression levels for individual genes were performed using the R package DESeq 2 with R version 3.5.0 [147].

### Motif Analysis

Peaks were called for each sample with replicates pooled, using the same method described above for individual replicates. Then, FIMO, from MEME version 4.11.2 [148], was used to scan for motif occurrences within 200 bp of the summit of each peak in each sample, separately. For cases, where multiple motifs were called for the same peak, only the most statistically significant motif was kept. To determine motif enrichment in the HD samples, we constructed a 2 × 2 contingency matrix for each motif separately, as follows. For example, for motif X, the matrix

$$ \begin{array}{*{20}c} {\text{N11}} & {\text{N12}} \\ {\text{N21}} & {\text{N22,}} \\ \end{array} $$

was constructed, where N11 = # of times motif X overlaps an HD-up differential peak, N12 = # the number of times all other motifs besides X overlap an HD-up differential peak, N21 = # of times motif X overlaps any HD peak, and N22 = # of times all motifs except X overlap any HD peak. Fisher’s exact test was then used to test the null hypothesis N11/(N11 + N12) = N21/(N21 + N22), i.e., that the proportion of times that motif X occurs in HD-up differential peaks is the same as the proportion amongst all HD peaks (i.e., what would be expected if a random sample of HD peaks were selected). Those motifs with low *p* values, therefore, have a much higher proportion of motif X occurring in HD-up differential peaks than would be expected by chance by randomly selecting HD peaks.

This procedure was performed separately for proximal-promoter peaks (TSS ± 500 bp) and for distal peaks (> 500 bp from any TSS). The same procedure was conducted for each motif using WT-up differential peaks and WT peaks to determine the significance of motif enrichment in WT samples. In that case, the same matrix as above was constructed, except with N11 = # of times the motif X overlaps a WT-up differential peak, N12 = # the number of times all other motifs besides X overlap a WT-up differential peak, N21 = # of times motif X overlaps any WT peak, and N22 = # of times all motifs except X overlap any WT peak.

*p* values from this test were used to determine the size of circles in the motif enrichment plots, as shown in Fig. 1f, g. No multiple test corrections were used in this case, because the high degree of degeneracy of motifs for different transcription factors means that the *p* values for numerous pairs of motifs are strongly dependent on one another, whereas multiple test corrections such as *q* value require that all *p* values have at most a weak degree of dependence [149]. A weak correlation amongst *p* values is hypothesized amongst, e.g., genome-wide differential expression analyses from RNA-seq [149], but in the case of a list of TF motifs, the relatively small number of TFs combined with the high degree of correlation of *p* values amongst related TFs mean that the dependency amongst *p* values are stronger than for *p* values from a genome-wide differential expression analysis.

### Gene set enrichment analysis (GSEA)

To identify pathways differentially modulated between WT and HD samples and different differentiation stages, GSEA [104] was performed as follows. For each contrast, transcripts were ranked by differential expression using the Signal2Noise metric. GSEA was performed using the desktop module available from the Broad Institute (http://www.broadinstitute.org/gsea/). GSEA was performed on the ranked transcript lists using 1000 gene set permutations, collapse of duplicates to Max probe, and random seeding. Gene sets used included the (H) Hallmark and (C5) GO_BP gene sets (MSigDB v6.2) [99].

## Supplementary information

**Additional file 1: Figure S3.** Related to Fig. 1. **Figure S4.** Related to Fig. 3. **Figure S5.** Related to Fig. 4. **Figure S6.** Related to Fig. 5. **Figure S7.** Related to Fig. 5. **Figure S8.** Related to Fig. 6. **Figure S9.** Related to Fig. 6. **Table S1.** RT-qPCR primers.

**Additional file 2: Table S2.** Differentially expressed genes across HD astrocyte differentiations.

**Additional file 3: Table S3.** 36 genes differentially expressed at every stage of HD astrocyte differentiation.

**Additional file 4: Table S4.** Summary of ATAC-seq data.

**Additional file 5: Table S5.** Motifs identified at differential ATAC-seq peaks during differentiation.

**Additional file 6: Table S6.** List of nearest genes to differential ATAC-seq peaks at each stage of differentiation.

**Additional file 7: Table S7.** Motifs enriched in nearest differential peaks associated with E2F target genes.

## Data Availability

All data sets have been deposited in the NCBI GEO database, with the Accession Number GSE130570. Reviewers can access the data using token ihojuwwejzuzlof.
