# Supplementary material for: Chromatin accessibility and transcription dynamics during in vitro astrocyte differentiation of Huntington’s Disease Monkey pluripotent stem cells
Source: Epigenetics Chromatin. 2019 Nov 13;12:67. doi: 10.1186/s13072-019-0313-6 (PMC6852955; doi:10.1186/s13072-019-0313-6)
Supplement: Supplementary file 1 — Additional file 1: Figure S3. Related to Fig. 1. Figure S4. Related to Fig. 3. Figure S5. Related to Fig. 4. Figure S6. Related to Fig. 5. Figure S7. Related to Fig. 5. Figure S8. Related to Fig. 6. Figure S9. Related to Fig. 6. Table S1. RT-qPCR primers. [file 13072_2019_313_MOESM1_ESM.pdf]

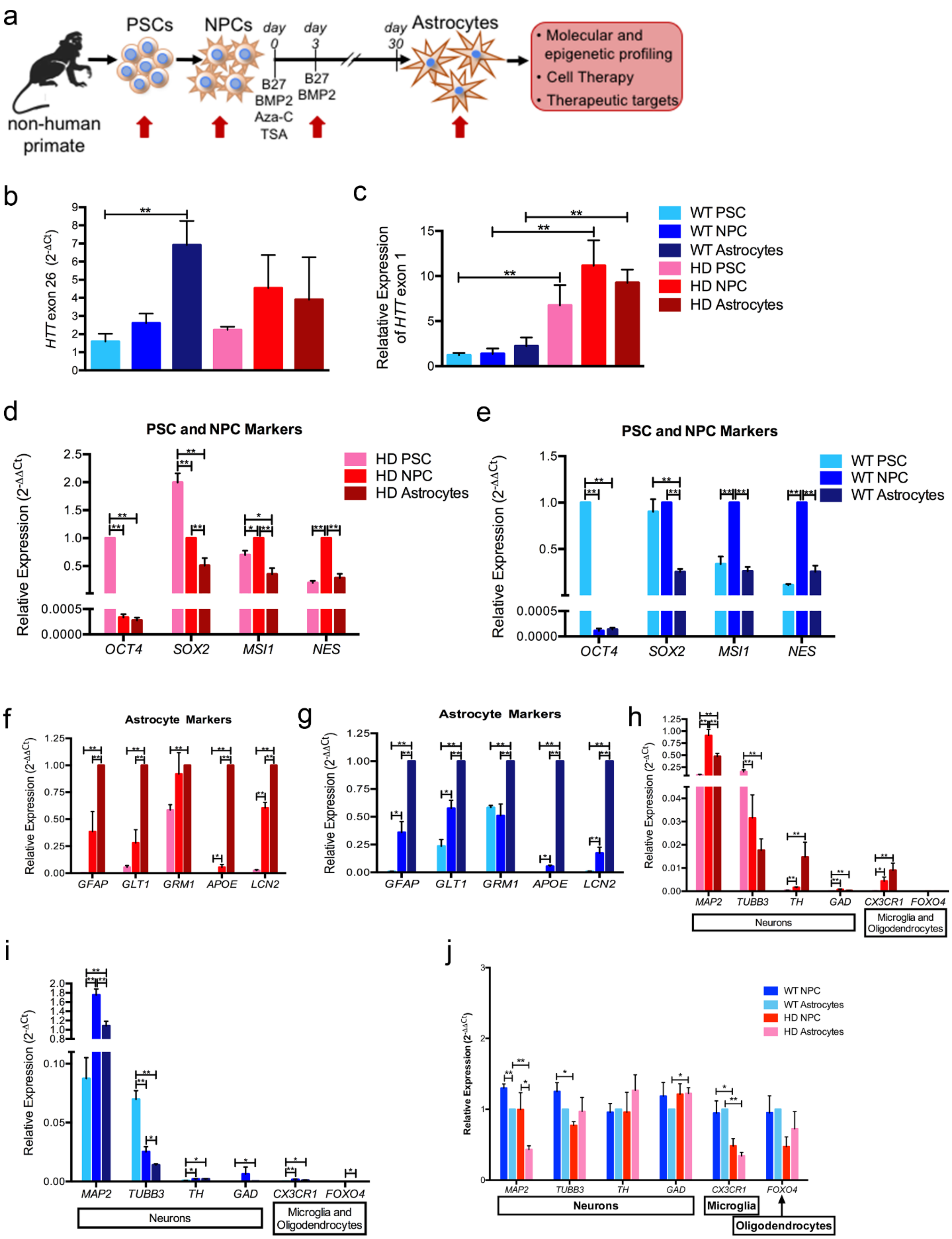

a
non-human primate
PSCs
NPCs
day 0
day 3
day 30
Astrocytes
B27
BMP2
Aza-C
TSA
B27
BMP2
• Molecular and epigenetic profiling
• Cell Therapy
• Therapeutic targets
b
HTT exon 26 (2^-ΔCt)
c
Relative Expression of HTT exon 1
WT PSC
WT NPC
WT Astrocytes
HD PSC
HD NPC
HD Astrocytes
d
PSC and NPC Markers
Relative Expression (2^-ΔΔCt)
HD PSC
HD NPC
HD Astrocytes
OCT4
SOX2
MSI1
NES
e
PSC and NPC Markers
WT PSC
WT NPC
WT Astrocytes
OCT4
SOX2
MSI1
NES
f
Astrocyte Markers
GFAP
GLT1
GRM1
APOE
LCN2
g
Astrocyte Markers
GFAP
GLT1
GRM1
APOE
LCN2
h
Relative Expression (2^-ΔCt)
MAP2
TUBB3
TH
GAD
CX3CR1
FOXO4
Neurons
Microglia and Oligodendrocytes
i
Relative Expression (2^-ΔCt)
MAP2
TUBB3
TH
GAD
CX3CR1
FOXO4
Neurons
Microglia and Oligodendrocytes
j
Relative Expression (2^-ΔΔCt)
WT NPC
WT Astrocytes
HD NPC
HD Astrocytes
MAP2
TUBB3
TH
GAD
CX3CR1
FOXO4
Neurons
Microglia
Oligodendrocytes

Figure S1. (a) Model of study design. Pluripotent stem cells (PSCs) were derived from HD and WT nonhuman primates (NHPs). Neural progenitor cells (NPCs) were further differentiated *in vitro* into astrocytes using a 30-day differentiation protocol. Time points for samples used in this study are indicated by red arrows. (b-c) qRT-PCR analysis of *HTT* exon 26 expression (b) and of *HTT* exon 1 expression relative to *HTT* exon 26 expression (c) during *in vitro* astrocyte differentiation of WT and HD NHP cell lines. Expression of both exons is normalized to *GAPDH* expression. (d-j) qRT-PCR analysis demonstrates differentiation efficiency. HD (d, f, h) and WT (e, g, i) NHP cells show appropriate expression of lineage specific markers, with decreases in PSC/NPC markers (d-e), increases in astrocyte markers (f-g), and repression of markers for other neural lineages (h-j) during astrocyte differentiation. All qRT-PCR experiments were performed in triplicate, using three biological replicates. Data and error bars are represented as mean ± SEM (**$p < 0.01$ and *$p < 0.05$, ANOVA).

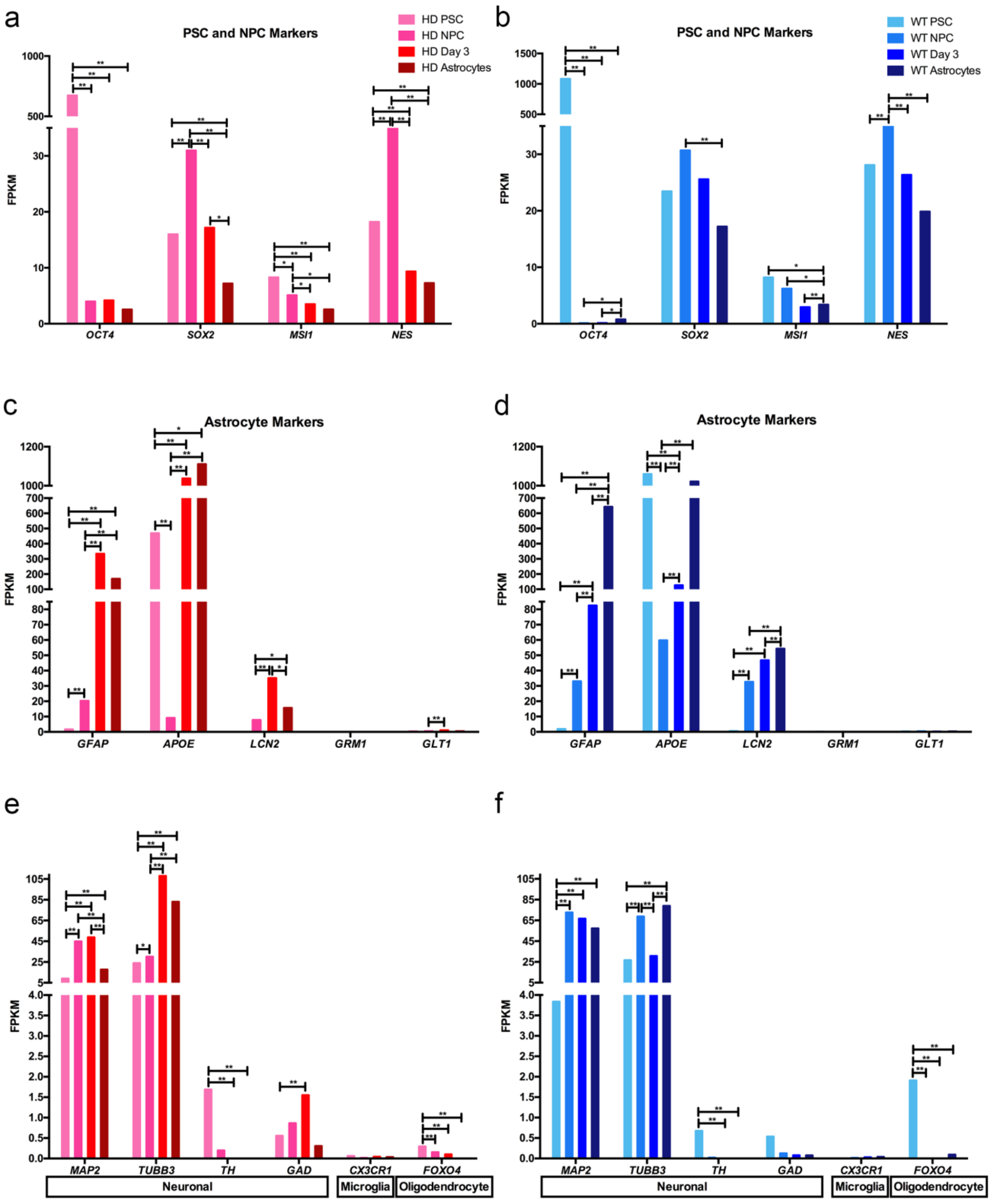

Figure S2. (a-f) Bar graphs of RNA-seq signal of cell-type markers during *in vitro* astrocyte differentiation in HD (a, c, e) and WT (b, d, f) cells. (a-b) PSC and NPC markers are downregulated

during differentiation. (c-d) Several astrocyte markers become induced during astrocyte differentiation. (e-f) Markers for neuronal and glial cell types are largely repressed over the course of astrocyte differentiation. All RNA-seq experiments were performed in three biological replicates and average FPKM for each sample was plotted (**$p < 0.001$ and *$p < 0.01$, differential expression analysis).

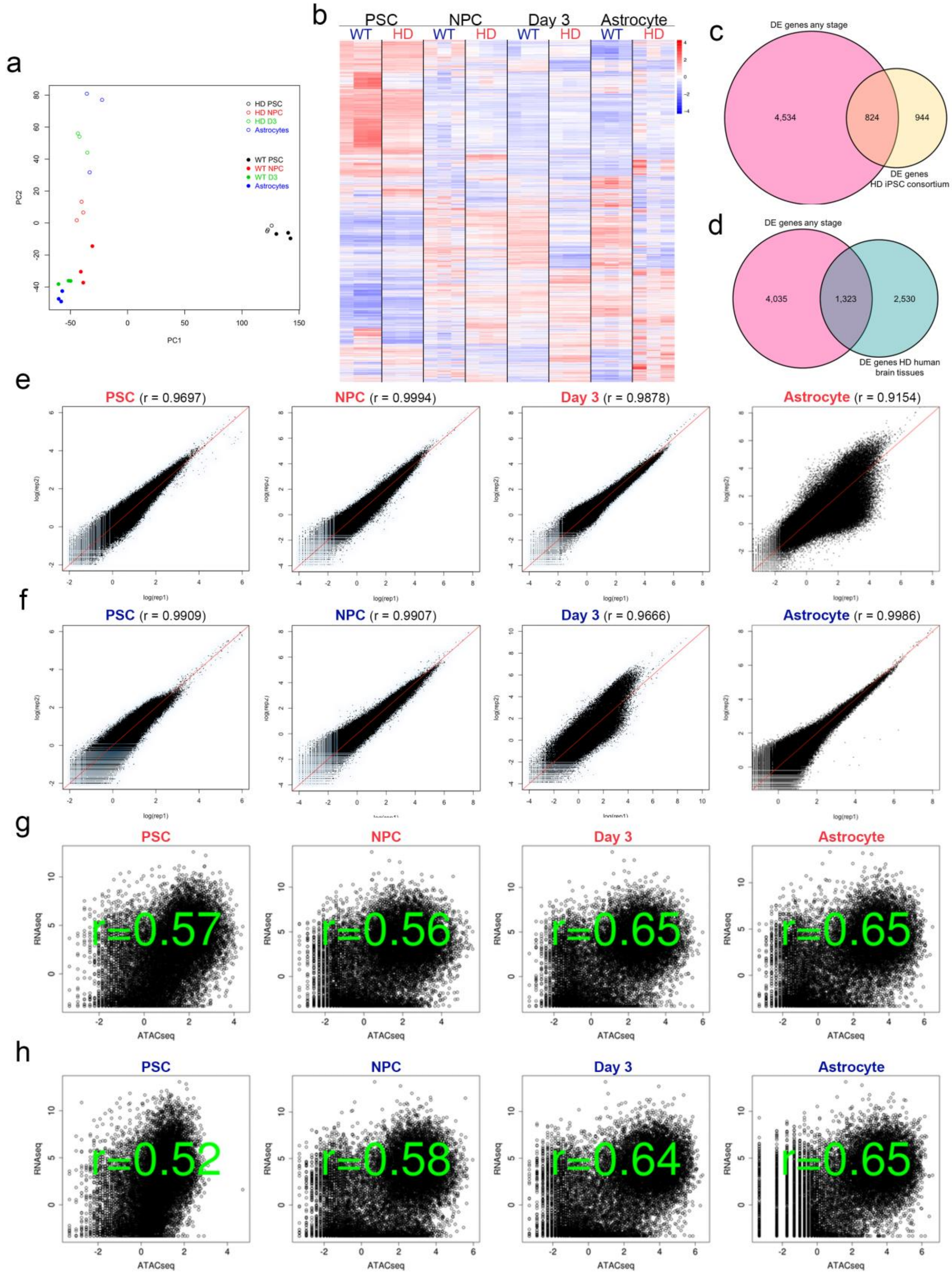
a
HD PSC
HD NPC
HD D3
Astrocytes
WT PSC
WT NPC
WT D3
Astrocytes
PC1
PC2
b
PSC
NPC
Day 3
Astrocyte
WT HD WT HD WT HD WT HD
c
DE genes any stage
4,534
824
944
DE genes
HD iPSC consortium
d
DE genes any stage
4,035
1,323
2,530
DE genes HD human
brain tissues
e
PSC (r = 0.9697)
NPC (r = 0.9994)
Day 3 (r = 0.9878)
Astrocyte (r = 0.9154)
f
PSC (r = 0.9909)
NPC (r = 0.9907)
Day 3 (r = 0.9666)
Astrocyte (r = 0.9986)
log(rep1)
log(rep2)
g
PSC
r=0.57
NPC
r=0.56
Day 3
r=0.65
Astrocyte
r=0.65
h
PSC
r=0.52
NPC
r=0.58
Day 3
r=0.64
Astrocyte
r=0.65
ATACseq
RNAseq

Figure S3. Related to Figure 1. (a) Principal component analysis (PCA) of log2 transformed normalized read counts (variance stabilizing transformation, DESeq2) from RNA-seq experiments in HD (outline) and WT (solid) cell lines for all 4 time points, PSC (black), NPC (red), Day 3 (green), and astrocyte (blue). PC1 and PC2 capture 51% and 13% of the data variance respectively. (b) Heatmap of $\log_2$ transformed normalized read counts (variance stabilizing transformation, DESeq2) of transcripts differentially expressed (FDR adjusted, alpha=0.02, n=5,062) between different differentiation stages and between WT and transgenic samples. Expression values of each transcript (rows) are scaled to unit variance across samples (columns) to normalize expression between transcripts. Transcripts are organized by unsupervised hierarchical clustering (euclidean distance, complete linkage) and samples are organized by differentiation stage and genotype (WT or HD). (c) Venn diagram showing overlap of differentially expressed (DE) genes identified at any stage of HD astrocyte differentiation and DE genes identified by the HD iPSC Consortium (2017). Only DE genes annotated in both species' genomes were counted. (d) Venn diagram of overlapping DE genes across HD differentiation and DE genes identified in post-mortem HD brain tissues by Labadorf et al. (2015). Only DE genes annotated in both species' genomes were counted. (e-f) Scatterplots between two ATAC-seq replicates for HD (e) and WT (f) cells at each stage of differentiation. (g-h) Scatterplots of RNA-seq gene FPKM values vs. ATAC-seq FPKM values at the promoters (TSS +/- 500bp) of the corresponding genes for HD (g) and WT (h) cells at each stage of differentiation. r = Pearson's correlation coefficient.

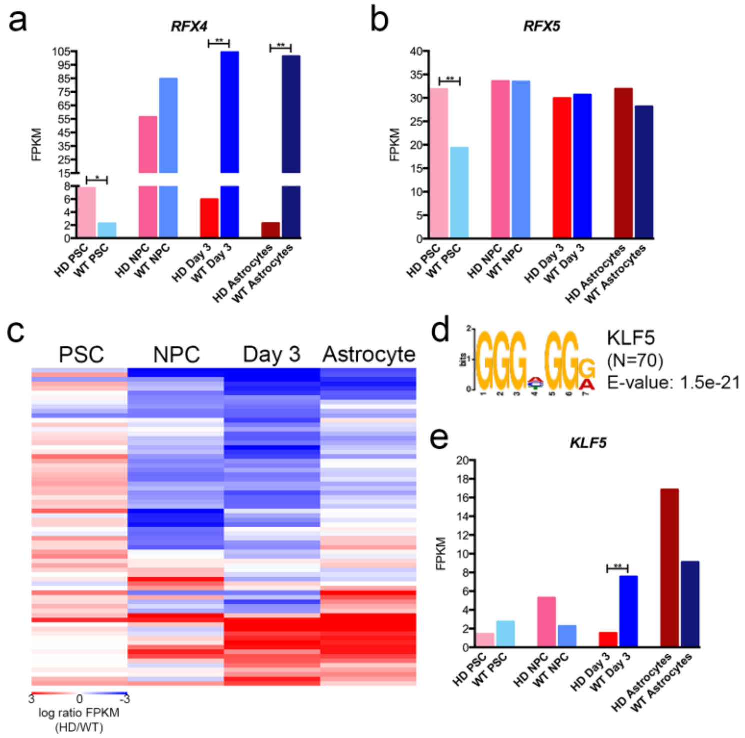

Figure S4. Related to Figure 3. (a-b) Bar graphs generated from RNA-seq data depicting differential expression of *RFX4* (a) and *RFX5* (b) occurs in at least one stage of *in vitro* astrocyte differentiation. RNA-seq experiments were performed in three biological replicates and average FPKM for each sample was plotted (**$p < 0.001$ and *$p < 0.01$, differential expression analysis). (c) Heatmap depicting normalized differential ATAC-seq signal in HD and WT cells at macaque brain enhancers overlapping a differential THSS that contains the KLF5 motif (N=70). The red color represents HD-enrichment and the blue color indicates WT-enrichment. (d) KLF5 motif found at enhancers that overlap a differential THSS. (e) Bar graph generated from RNA-seq data of *KLF5* expression across *in vitro* astrocyte differentiation. RNA-seq experiments were performed in three biological replicates and average FPKM for each sample was plotted (**$p < 0.001$ and *$p < 0.01$, differential expression analysis).

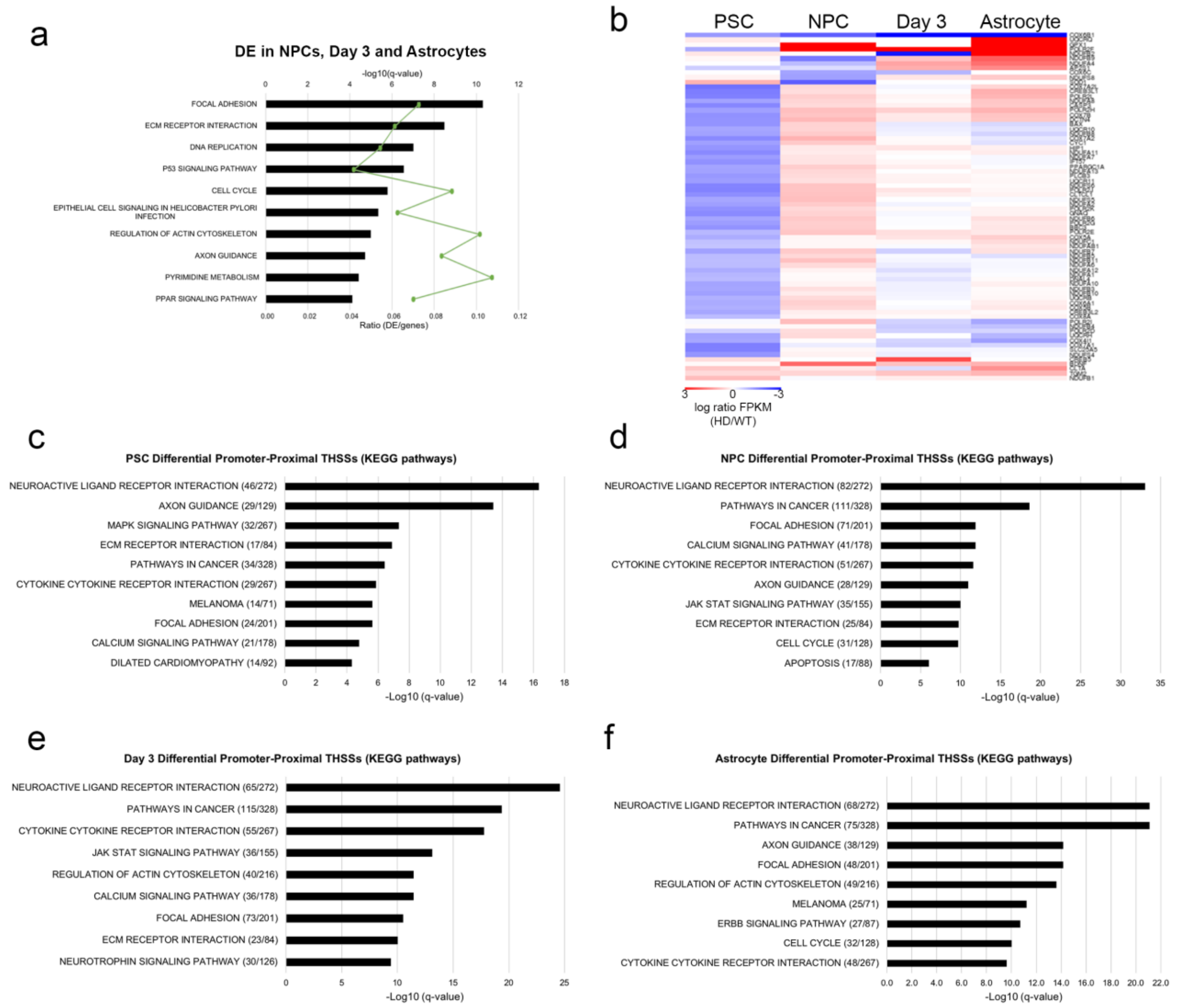

Figure S5. Related to Figure 4. (a) GO analyses of DE genes at the NPC, day 3 and astrocyte stages. Pathways are ranked by -log10(q-value), determined by the Benjamini-Hochberg procedure. All pathways shown satisfy q < 0.05. Line graphs show the ratio of DE genes in each KEGG pathway. (b) Heatmap depicting DE genes in the KEGG Huntington pathway (N=74) at each stage. PSCs show a different pattern of dysregulation compared to the other stages of HD astrocyte differentiation. The red color represents genes that are upregulated in HD and the blue color represents genes that are downregulated in HD. Each row corresponds to the same gene. (c-f) GO analysis of the closest gene to each differential THSS. (c), NPC (d), day 3 (e) and astrocyte (f) stages, with KEGG pathways using GO analysis. The top 10 significant pathways are ranked by -log10(q-value), determined by Benjamini-Hochberg procedures; all pathways shown satisfy q < 0.05.

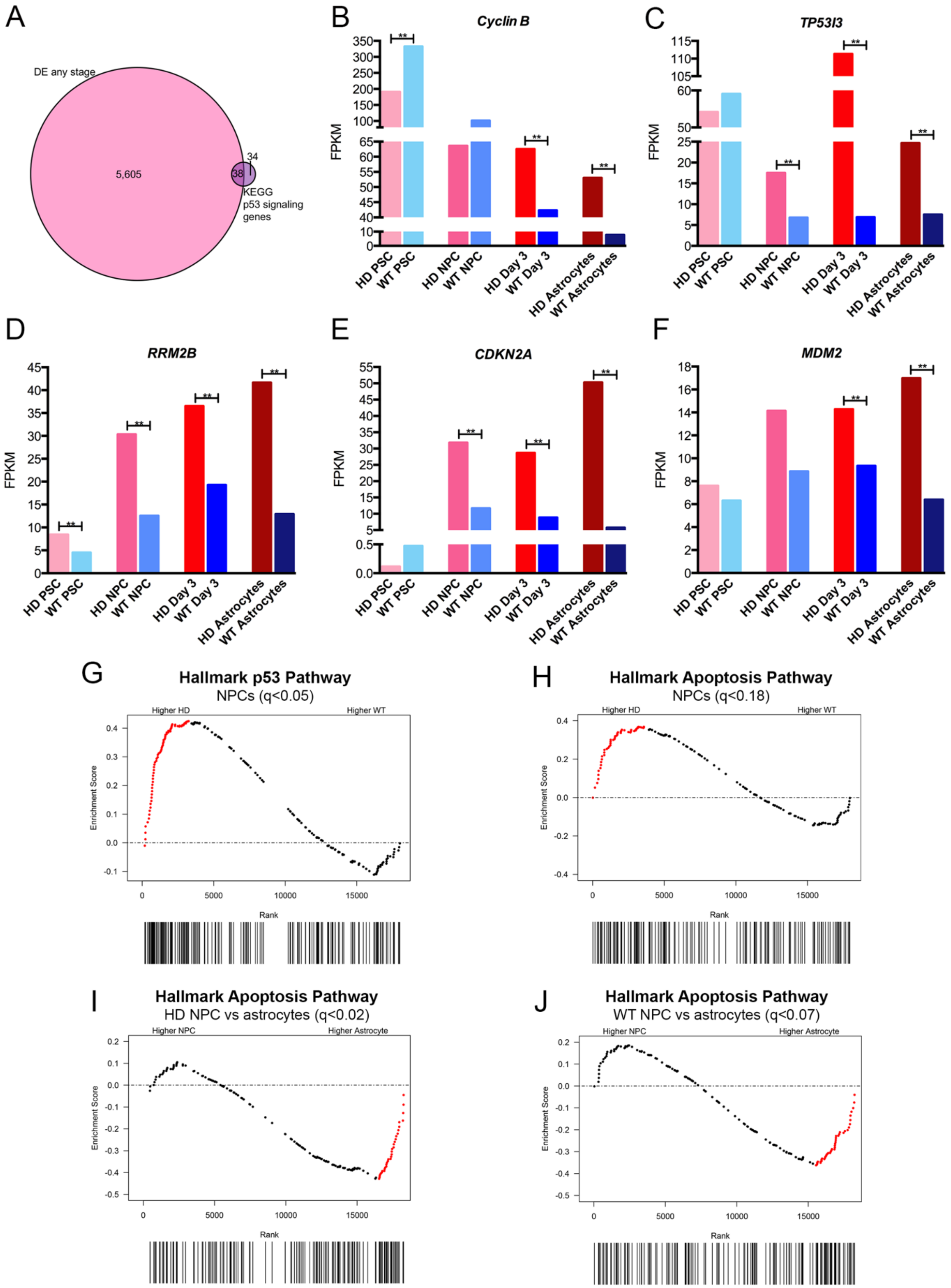

A
DE any stage
5,605
38
34
KEGG p53 signaling genes
B
Cyclin B
FPKM
HD PSC
WT PSC
HD NPC
WT NPC
HD Day 3
WT Day 3
HD Astrocytes
WT Astrocytes
C
TP53I3
D
RRM2B
E
CDKN2A
F
MDM2
G
Hallmark p53 Pathway
NPCs (q<0.05)
Higher HD
Higher WT
Enrichment Score
Rank
H
Hallmark Apoptosis Pathway
NPCs (q<0.18)
I
Hallmark Apoptosis Pathway
HD NPC vs astrocytes (q<0.02)
Higher NPC
Higher Astrocyte
J
Hallmark Apoptosis Pathway
WT NPC vs astrocytes (q<0.07)

Figure S6. Related to Figure 5. (a) Venn diagram showing overlap of DE genes at any stage with the KEGG p53 signaling pathway gene set. (b-f) Bar graphs of RNA-seq data for example DE genes in the p53 pathway. Average FPKM for each sample was plotted (**$p < 0.001$ and *$p < 0.01$, differential expression analysis). (g-j) GSEA enrichment plots. Cross sectional GSEA displays no significant enrichment of p53 signaling (g) or apoptosis (h) gene expression in HD NPCs. (i-j) Longitudinal GSEA of DE genes reveals significant enrichment of apoptosis pathway genes in HD astrocytes compared to HD NPCs (i), but not in WT cell lines (j). Red dots indicate leading edge genes. q-values are FDR corrected p-values with alpha=0.02, or the equivalent.

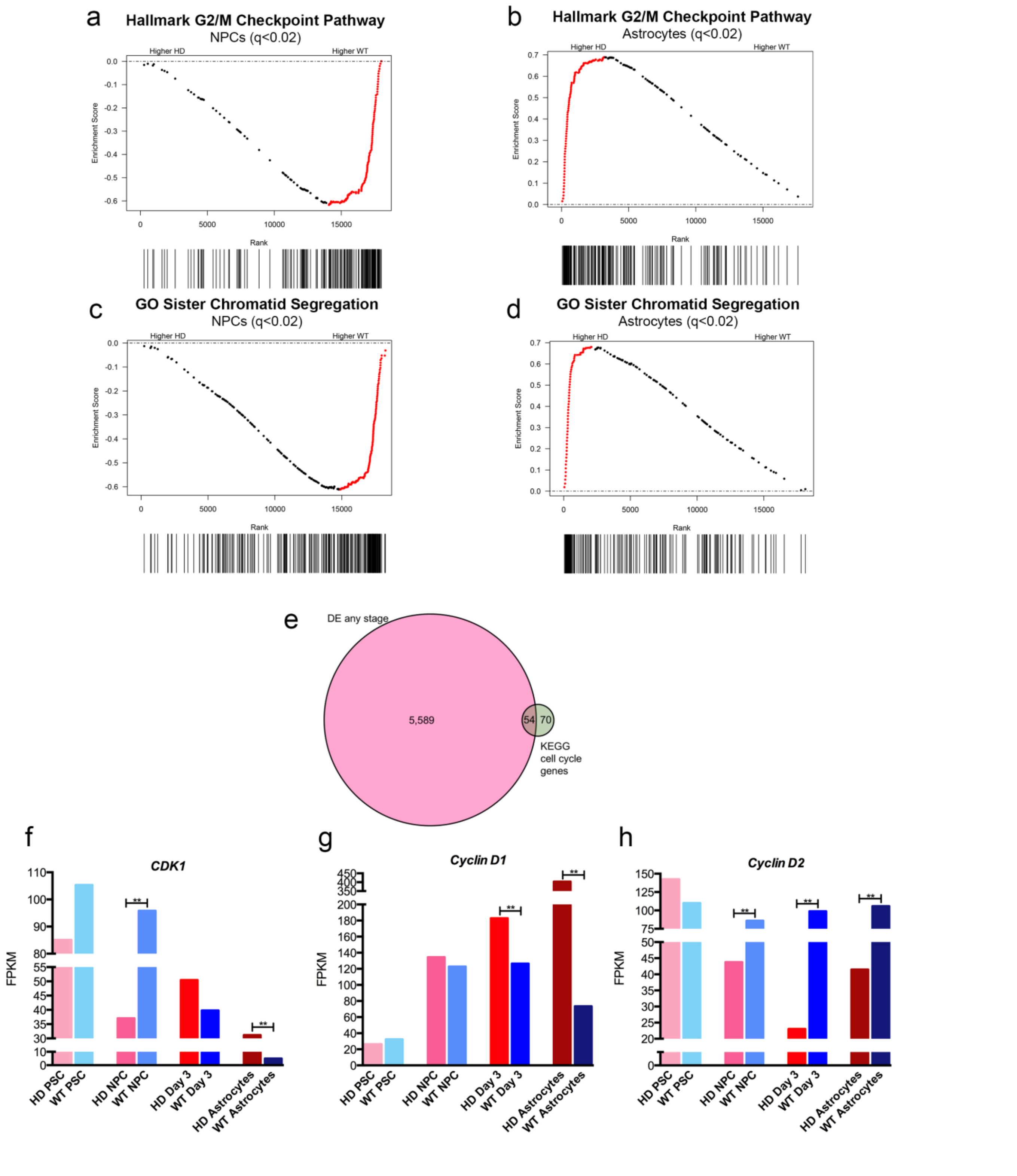

Figure S7. Related to Figure 5. (a-d) Cross sectional GSEA revealed an inverse enrichment of cell cycle pathways during HD astrocyte differentiation. (a-b) GSEA of DE genes revealed

depleted expression of G2/M checkpoint genes in HD NPCs (a) and enriched expression in HD astrocytes (b). (c-d) Genes involved in sister chromatid segregation are also inversely enriched in our DE gene set, with depletion in HD NPCs (c) and enrichment in HD astrocytes (d) compared to WT cells. Black lines indicate the position of pathway genes in rank-sorted expression data from HD and WT samples. Red dots indicate leading edge genes. q-values are FDR corrected p-values with alpha=0.02, or the equivalent. (e) Venn diagram showing overlap of DE genes at any stage with the KEGG cell cycle pathway gene set. All KEGG cell cycle pathway genes are counted, even those not annotated in MacaM. (f-h) Example cell cycle genes showing differential expression via RNA-seq. Average FPKM for each sample was plotted (**p < 0.001 and *p < 0.01, differential expression analysis).

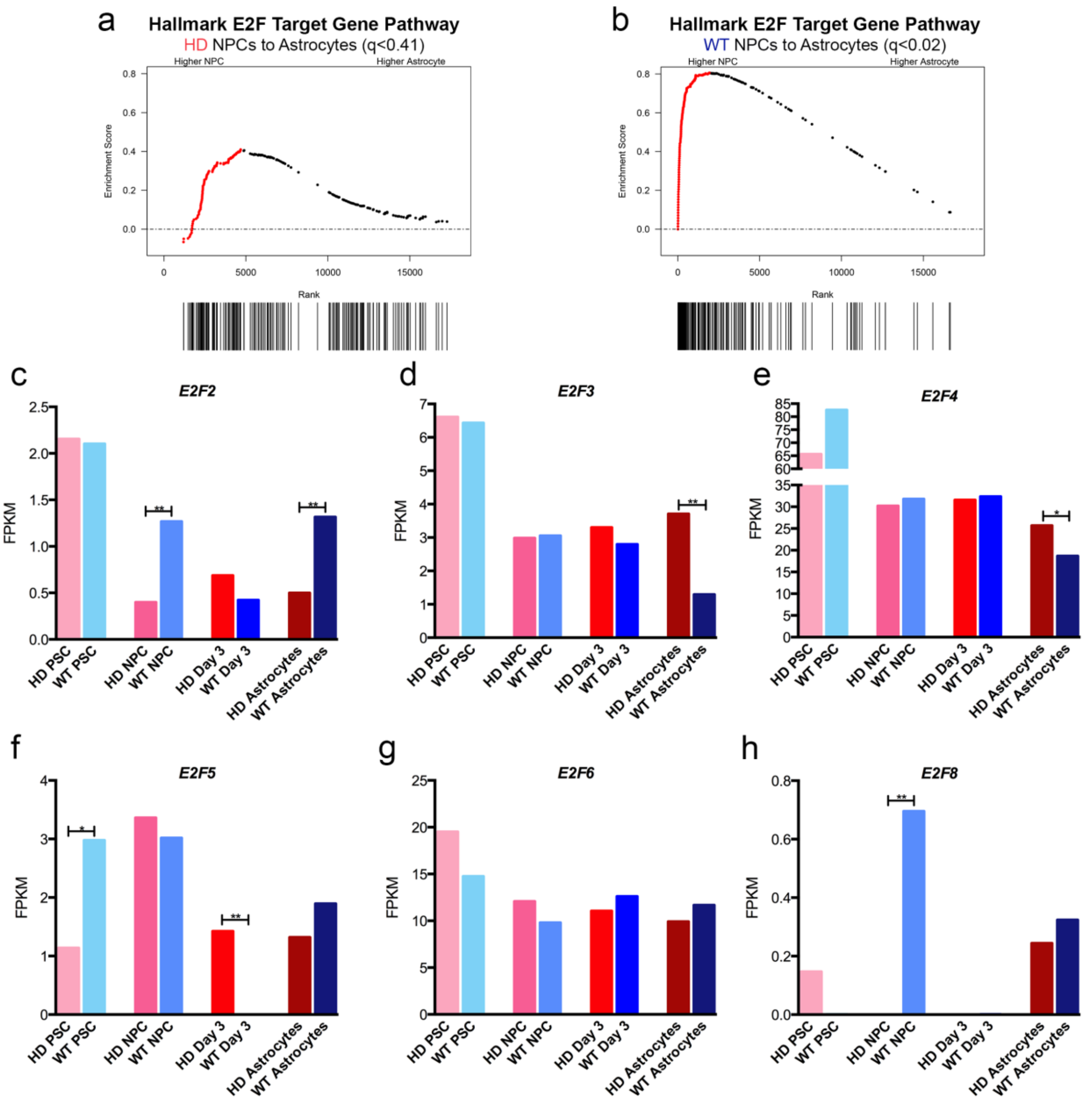

Figure S8. Related to Figure 6. (a-b) Longitudinal GSEA revealed enrichment of E2F target genes in WT (b) but not HD (a) NPCs. Black lines indicate the position of pathway genes in rank-sorted expression data from HD and WT samples. Red dots indicate leading edge genes. q-values are FDR corrected p-values with alpha=0.02, or the equivalent. (c-h) Bar graphs generated from RNA-seq data depicting differential expression of E2F family members, *E2F2* (c), *E2F3* (d), *E2F4* (e), *E2F5* (f), *E2F6* (g), *E2F8* (h). RNA-seq experiments were performed in three biological replicates and average FPKM for each sample was plotted (**p < 0.001 and *p < 0.01, differential expression analysis).

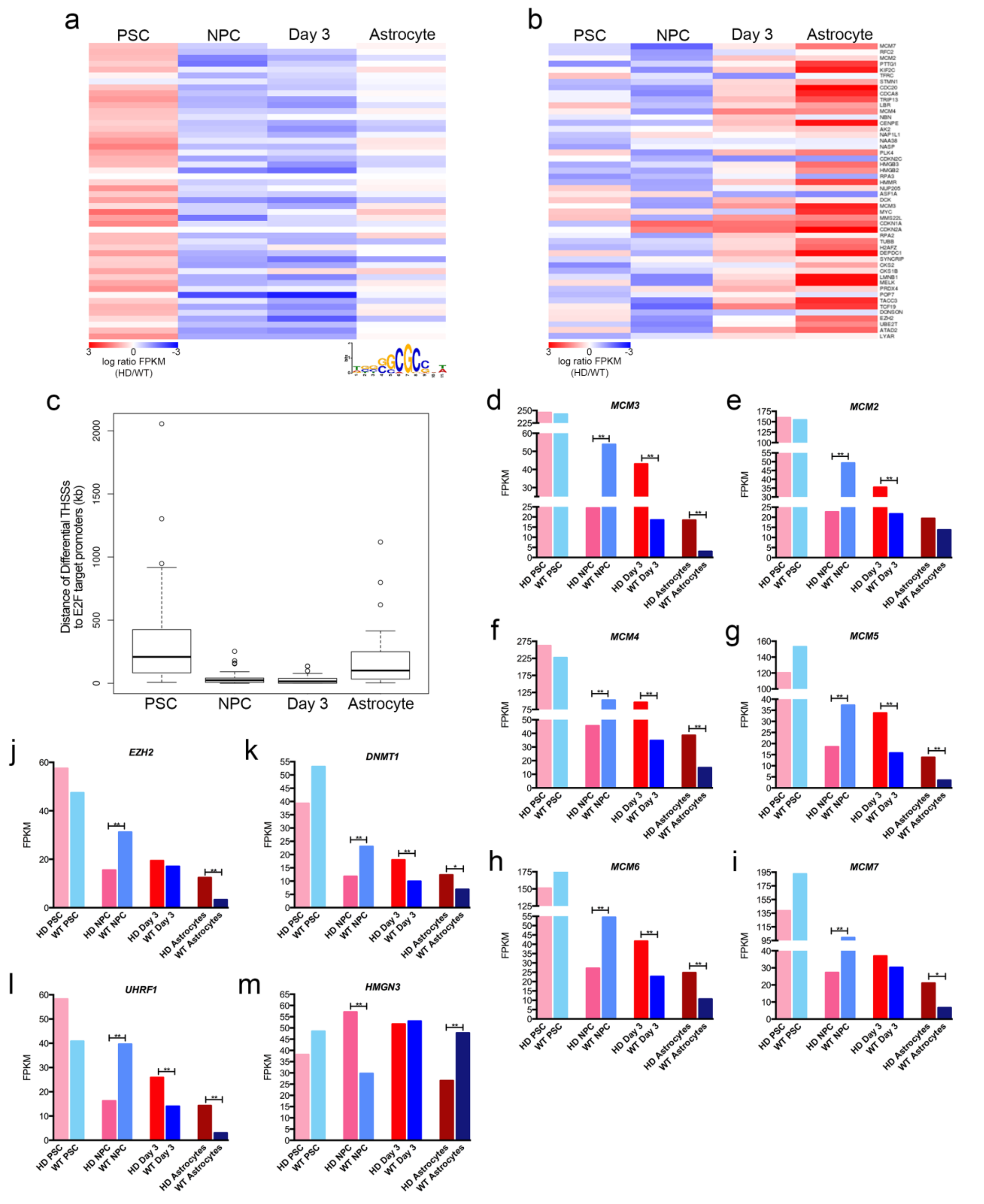

Figure S9. Related to Figure 6. (a) Heatmap depicting differential ATAC-seq signal at DE E2F target gene promoters shows depleted accessibility in HD NPCs and at day 3, while HD and WT astrocytes display similar E2F target promoter accessibility. Promoter-proximal ATAC-seq peaks

are arranged according to the order of the genes in panel B. The E2F1 motif is shown below the heatmap. (b) Heatmap of differential E2F target gene expression at each stage of differentiation. The same heatmap is shown in Figure 6 but is included here for comparison with panel a. Gene names are displayed to the right of the plot. For both heat maps, red represents HD enrichment and blue indicates HD depletion. (c) Boxplot of the distribution of distances from each DE E2F gene promoter to the nearest differential THSS at each stage across astrocyte differentiation. (d-i) RNA-seq data for all six MCM proteins regulated by E2F TFs; *MCM3* (d), *MCM2* (e), *MCM4* (f), *MCM5* (g), *MCM6* (h), *MCM7* (i), all of which showed DE in at least one stage of differentiation. (j-m) Inverse DE profiles for several epigenetic regulators that are E2F target genes; *EZH2* (j), *DNMT1* (k), *UHRF1* (l) and *HMGN3* (m). RNA-seq experiments were performed in three biological replicates and average FPKM for each sample was plotted (**$p < 0.001$ and *$p < 0.01$, differential expression analysis).

Supplementary Table 1. RT-qPCR primers

Gene Symbol	SYBR Forward Primer	SYBR Reverse Primer
HTT Exon 1	GCGACCCTGGAAAAGCTGAT	CTGCTGCTGCTGGAAGGACT
HTT Exon 26	ACCCTGCTCTCGTCAGCTTGG	AGCAAGTTTCCGGCCAAAAT
SOX2	CACAGCGCCCGCATGTACAA	AGTTCGCTGTCCTGCCCTCA
MSI1	CACAGCCCAAGATGGTGACT	TCCACCTTCCCAAACTGCTC
NES	TGGCAAGAGGCCGGTACA	CCGTATTTGTCCTTCACCTTC
GFAP	CCAGCTCGCGGTTCTCATAC	CTCATGGACTTTCAGGGCGT
GLT1	ATGCACGACAGTCACCTCAG	AGGATGACACCAAACACCGT
GRM1	CTCGGGCATGCATTGTGAAA	GCGTTCTTGTTAGCAGTCCC
APOE	GGGTCGCTTTTGGGATTACC	CTCATCCATCAGCGTCGTCA
LCN2	AGGGAATGCAGTTGGCAGAA	GGAGGTCACGTTGTAGCTCT
MAP2	ATCTTTCTCCTCTGGCTTCCG	GGTGTGGTGGCTGGAAGGTA
TUBB3	GCCAAGTTCTGGGAAGTCAT	GGCACGTACTTGTGAGAGGA
TH	GAACTTCTGGGGTCGCTCC	ACCTCAAGACTTACCGGCTT
GAD	CCACGTTTTTGGCGAACG	CAGTGTCGCTTTTTCGGTGT
CX3CR1	AAAACGAATGCCTTGGTGAC	AGGAAAAACACGACGACCAC
FOXO4	ACCATGGATGTGTTAGGGGC	CCCTGTGTGTAAATGGGGGA
UBC	CCACTCTGCACTTGGTCCTG	CCAGTTGGGAATGCAACAACTTTA
	**TaqMan® Primer Context Sequence**	
OCT4	CCCTGGGGGTTCTATTTGGGAAGGT	
UBC	TCCTTTCAATAAAGTTGTTGCATTC	
